# The Role of the Bone Marrow Microenvironment in the Pathogenesis of Acute Myeloid Leukemia

**DOI:** 10.3390/biomedicines14081679

**Published:** 2026-07-27

**Authors:** Michele Gottardi, Federico De Marchi, Giulia Ciotti, Marco Basso, Vittoria Raimondi, Vincenzo Ciminale, Giorgia Simonetti, Martina Ghetti, Rosa Di Liddo, Roberta De Marchi, Islam Ab Abouzeid, Alessandra Sperotto

**Affiliations:** 1 Onco-Hematology, Department of Oncology, Veneto Institute of Oncology (IOV-IRCCS), 31033 Padua, Italy; federico.demarchi@iov.veneto.it (F.D.M.); giulia.ciotti@iov.veneto.it (G.C.); roberta.demarchi@iov.veneto.it (R.D.M.); alessandra.sperotto@iov.veneto.it (A.S.); 2 Pharmacy, Veneto Institute of Oncology (IOV-IRCCS), 31033 Padua, Italy; marco.basso@iov.veneto.it; 3 Immunology and Molecular Oncology Unit, Veneto Institute of Oncology (IOV-IRCCS), 31033 Padova, Italy; vittoria.raimondi@iov.veneto.it (V.R.); vincenzo.ciminale@iov.veneto.it (V.C.); 4 Biosciences Laboratory, IRCCS Istituto Romagnolo per Lo Studio Dei Tumori (IRST) “Dino Amadori”, 47014 Meldola, Italy; giorgia.simonetti@irst.emr.it (G.S.);; 5 Department of Pharmaceutical and Pharmacological Sciences, University of Padova, 35131 Padova, Italy; rosa.diliddo@unipd.it; 6 School of Medicine and Surgery, University of Padova, 35131 Padova, Italy; islamabdelhamidbashir.abouzeid@studenti.unipd.it

**Keywords:** bone marrow microenvironment, acute myeloid leukemia, leukemia stem cell

## Abstract

Acute myeloid leukemia (AML) develops within a bone marrow environment that influences leukemic stem cell behavior, residual disease, and response to therapy. This review examines evidence that the marrow microenvironment is not only a site of leukemic growth, but can actively shape AML initiation, maintenance, and treatment resistance. Clinical observations such as donor cell leukemia after allogeneic transplantation, together with experimental models in which stromal or osteolineage abnormalities induce myeloid disease, suggest that altered niches may contribute to leukemogenesis in selected settings. In established AML, vascular and endosteal compartments provide adhesive, chemokine, inflammatory, and metabolic signals that promote leukemic-cell retention, quiescence, survival, and chemotherapy tolerance. AML cells also remodel the surrounding marrow, suppressing normal hematopoiesis and generating stromal, endothelial, osteoblastic, adipocytic, and immune-cell programs that favor leukemic persistence. These interactions are especially relevant to drug resistance, including resistance to venetoclax-based therapy, where cytokine-mediated changes in apoptotic dependence, fatty-acid metabolism, mitochondrial adaptation, and stromal support may all contribute. Several therapeutic approaches have attempted to disrupt niche-mediated protection, including targeting CXCL12/CXCR4 signaling, adhesion pathways, inflammatory circuits, Hedgehog signaling, and metabolic dependencies. Although early-phase studies have shown activity in some AML subsets, randomized evidence remains limited and results have been inconsistent. We discuss how a better understanding of microenvironmental biology may help define when niche-directed therapy is most likely to complement conventional and molecularly targeted AML treatment.

## 1. Introduction

Darwin observed that “a grain in the balance will determine which individual shall live or die, which species will increase or become extinct” [1]. Evolution proceeds through small, random variations, of which only a few prove advantageous: the surviving species is the one that adapts most favorably to its environment.

In the 1950s and 1960s, Till and McCulloch proposed a hierarchical, pyramidal organization of hematopoiesis, with a self-renewing, asymmetrically dividing hematopoietic stem cell (HSC) at its apex [2]. Asymmetric mitosis allows the cell both to differentiate toward a specific function and to self-regenerate at the top of the pyramid, maintaining the stem pool in quiescence and ensuring tissue inexhaustibility. The same logic applies to neoplastic tissues: relapse after morphological remission implies persistence of a quiescent, chemoresistant cancer stem cell with indefinite self-renewal, which drives carcinogenesis [3].

Stemness is defined functionally rather than by surface phenotype, as the capacity to regenerate the tissue of origin through serial xenotransplantation into immunodeficient hosts [4]. A stem cell—normal or leukemic—is conventionally defined by its capacity to sustain tissue in at least three serial transplants; hence the terms leukemic stem cell (LSC) or leukemia-initiating cell (L-IC). The observation that LSCs share the CD34^+^/CD38^−^ immunophenotype of HSCs led to the hypothesis that acute myeloid leukemia (AML) arises from oncogenic transformation of a normal HSC [5].

Immunophenotypic studies of LSCs began to disclose a role for the bone marrow microenvironment (BMM) in AML pathogenesis. In NOD/SCID/IL2Rγc-deficient models, lacking B, T, and NK cells [6], even committed progenitors expressing CD38 or CD45RA can regenerate AML in serial transplants, fulfilling LSC criteria [7]. In a sufficiently permissive microenvironment, stemness is therefore not restricted to primitive cells.

These data reframe malignancy: aggressiveness depends not only on genetic and epigenetic traits but on the tumor microenvironment that shapes proliferative advantage. A neoplasm is an ecosystem in which malignant cells interact with non-neoplastic components that modulate tumor fitness and response to therapy [8], honoring Darwin’s intuition at a microscopic scale.

The bone marrow is the physical setting in which this selection plays out. Its microenvironment comprises mesenchymal stromal cells and their osteoblastic and adipocytic progeny, sinusoidal and arteriolar endothelial cells, perivascular pericytes, sympathetic nerve fibers, and several immune populations. These cells are organized around two connected compartments: an endosteal, osteoblast-lined niche and a central, perivascular niche [9,10]. In normal hematopoiesis they govern the quiescence, self-renewal, retention, and mobilization of hematopoietic stem cells; in leukemia, the same signals can be redirected to shelter and sustain the malignant clone [11,12,13,14]. This review examines how the bone marrow microenvironment participates in AML along three connected lines. The first is its possible contribution to leukemic initiation; the second, the vascular and osteoblastic mechanisms that maintain leukemic stem cells and confer chemoresistance; the third, the reciprocal remodeling through which leukemic cells reshape the niche. We then consider how these mechanisms are being translated into microenvironment-directed therapies and where the clinical evidence currently stands.

## 2. Clues from Clinical Experience

Allogeneic hematopoietic stem-cell transplantation (HSCT) is a curative treatment for eligible patients with high-risk AML. Sustained remission depends on the graft-versus-leukemia (GVL) effect exerted by donor immunity against residual leukemia stem cells (LSCs) [15]. Relapse—the principal obstacle to cure—still occurs in 30–40% of transplanted patients [16]. Most relapses are of host origin; donor cell leukemia (DCL), in which leukemia arises de novo from engrafted donor hematopoiesis, is rare but mechanistically informative.

Registry data estimate DCL at 0.08% (European Society for Blood and Marrow Transplantation, EBMT) and 0.16% (Japan Society for Hematopoietic Cell Transplantation, JSHCT) across indications [17,18]. Among AML recipients, the reported incidence approaches 2%, with individual series describing rates up to 4–6%, and with a clear preponderance in adults [19]. DCL develops after a median of 28 months post-HSCT, although the range is wide (1 month to 24 years). The first described case (1971) involved a 16-year-old female with acute lymphoblastic leukemia (ALL) who, after transplantation from her brother, developed donor-derived ALL in XY hematopoietic cells [19].

Diagnostic sensitivity has improved with chimerism analysis by short-tandem-repeat (STR) and variable-number-tandem-repeat (VNTR) profiling. The pathogenesis is multifactorial, reflecting donor, recipient, and procedure-related contributions in a multiple-hit model.

Prior to 2010, cytogenetic analysis of DCL identified chromosome 7 abnormalities in 23% of cases versus ≤5% of de novo AML [20]—a distribution reminiscent of therapy-related myeloid neoplasms and consistent with persistent mutagenic effects of recipient-directed chemotherapy and conditioning on the marrow stroma. Next-generation sequencing has refined this picture. Among the transcription factors recurrently mutated in DCL—CEBPA, GATA2, RUNX1, and JAK2—three are typically of germline origin and one is predominantly somatic [20]. Donor clonal hematopoiesis, particularly DNMT3A-mutated clonal hematopoiesis of indeterminate potential (CHIP), has been documented in recipients who later developed DCL [21,22]; in related donors aged ≥50 years, CHIP prevalence reaches 16% [23]. Additional recurrently mutated genes cluster into splicing regulators (DDX41), epigenetic modifiers (ASXL1, DNMT3A, EZH2, IDH1/2), and DNA-repair genes (CHEK1, XPD, XRCC3) [20].

That donors do not typically develop AML, except in extremely rare cases, suggests that the recipient microenvironment plays a significant role in supporting leukemogenesis. Preclinical data link alterations in bone marrow stromal cells and other niche components to the development and maintenance of leukemia and to chemotherapy resistance [24,25,26,27,28]. Aldoss et al. reported two sequential myeloid DCLs in a single recipient after two HSCTs from different donors—a matched-related transplant followed three years later by a first DCL, then, after a new induction, a second transplant from a young unrelated donor with subsequent development of a second DCL from the second donor. The authors concluded that the recipient likely had a leukemogenic microenvironment that predisposed donor cells to transform [29,30].

DCL after cord-blood transplant (CBT) occurs earlier (median 14.5 vs. 36 months) and is more frequently associated with chromosome 7 abnormalities. Fusion genes such as TEL-AML1 or AML1-ETO may be present in cord cells and predispose to expansion of a pre-leukemic clone; these are class 2 genetic alterations that impair differentiation but do not alone induce overt AML, and they often occur years before disease onset. Because cord blood cells are less likely to have acquired predisposing genetic mutations, DCLs after CBT provide a compelling argument for a role of the microenvironment in initiating leukemic transformation.

## 3. Possible Direct Oncogenic Role of the Bone Marrow Microenvironment

Mesenchymal stromal cells (MSCs) sustain marrow homeostasis by regulating HSC self-renewal, differentiation, and immune tolerance. Primary MSCs from patients with myelodysplastic syndromes (MDS) and AML differ structurally, genetically, and functionally from healthy controls; dysregulation of KIT-ligand, angiopoietin-1, and Jagged1 signaling is associated with impaired osteogenic commitment and reduced growth capacity [9,31,32,33]. TGF-β signaling has been implicated as an important contributor to the aberrant MSC phenotype in both MDS and AML [34]. While the role of the BMM in disease maintenance and chemoresistance is increasingly recognized, its contribution to disease initiation is only now emerging.

Raaijmakers et al. deleted Dicer1—a ribonuclease III endonuclease indispensable for microRNA biogenesis—in murine MSCs at the osteolineage differentiation stage [26]. Osteolineage-specific loss disrupted osteoblastic differentiation without affecting the osteoclastic component, induced severe cytopenias with marked myelodysplastic features, and accelerated progression to acute leukemia. Transplantation of hematopoietic cells from Dicer1-mutant mice into wild-type recipients restored normal hematopoiesis, and the emergent leukemic clone retained wild-type Dicer1—demonstrating that the initiating event lay within the altered marrow microenvironment, not the hematopoietic compartment. Dicer1 inactivation in osteoprogenitors was also linked to downregulation of the Shwachman-Bodian-Diamond (Sbds) gene, which is involved in ribosomal biogenesis and whose germline loss produces Shwachman-Bodian-Diamond syndrome—characterized by skeletal alterations, inefficient hematopoiesis with bone marrow failure, and increased propensity to progress to MDS and AML. Reduced expression of Dicer1, Drosha, and Sbds has since been confirmed in MSCs from MDS and AML patients, together with loss of miR-155, miR-181a, and miR-222—microRNAs that regulate hematopoiesis [35,36,37].

Activating β-catenin mutations in murine osteoblasts reproduce an analogous niche-initiated leukemia [38]. β-catenin activation drives osteoblastic Jagged1 expression cooperatively with FoxO1, engaging Notch signaling in adjacent hematopoietic progenitors and producing AML with recurrent karyotypic alterations and autonomous progression [39]. Notch activation in this setting is associated with chemoresistance.

Epigenetic dysregulation also contributes to the altered functional state of the BMM. In primary stromal cells from MDS patients, aberrant cytosine hypermethylation silences FRZB, a WNT antagonist, thereby activating the WNT/β-catenin pathway in surrounding HSCs [40]. WNT activity affects HSC homeostasis in a dose-dependent manner, and increased WNT/β-catenin signaling has been linked to leukemic transformation [41,42]. Whether this stromal methylation defect precedes emergence of the myelodysplastic clone remains unresolved.

Cytotoxic therapy imprints a durable pro-inflammatory state on the MSC compartment. Alkylating agents induce cellular senescence and a senescence-associated secretory phenotype (SASP) with elevated pro-inflammatory cytokines and reactive oxygen species [43]. Multi-omic profiling of MSCs from patients with therapy-related myeloid neoplasms (t-MN) confirms induction of CDKN1A and β-galactosidase, FOS repression, elevated cytokine output, and impaired proliferation—a senescent-secretory signature that is stable regardless of the latency between cytotoxic exposure and t-MN diagnosis [44]. Analogous changes are seen in MSCs from patients with Fanconi anemia (FA), an inherited disease characterized by congenital abnormalities, bone marrow failure, and increased susceptibility to myeloid malignancies: baseline downregulation of FLT3-ligand, TGF-β1, and CXCL12, with cytoplasmic mislocalization of Dicer1 even before etoposide exposure. In healthy-donor MSCs, etoposide redistributes Dicer1 to the cytoplasm and induces ER-stress genes (GADD34, ATF4, NUPR1, HSPB), phenocopying the FA baseline [45]. Taken together, these data indicate that microenvironmental alterations may contribute to the pathogenesis of myeloid neoplasms, including AML, and suggest that they may also serve as therapeutic targets.

## 4. The Medullary Vascular Niche

**Figure 1:**
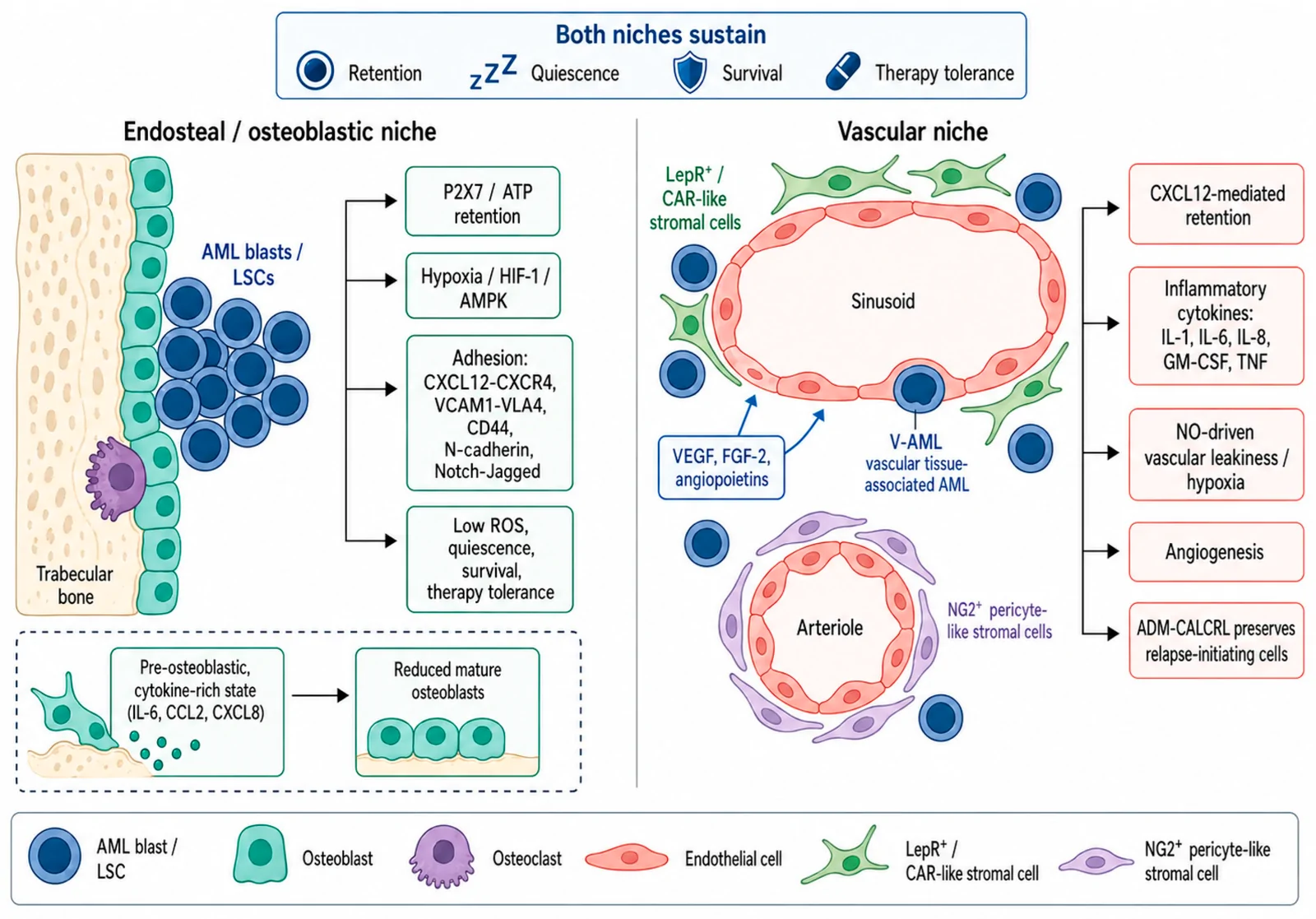
Bone marrow niches that sustain AML and their reciprocal remodeling. The endosteal/osteoblastic niche (**left**) retains LSCs through P2X7/ATP signaling, hypoxia–HIF-1α–AMPK metabolic reprogramming, and adhesion via CXCL12/CXCR4, VCAM-1/VLA-4, CD44 and N-cadherin, generating a low-ROS, drug-tolerant state; AML blasts in turn arrest osteoblast differentiation at a pre-osteoblastic stage with elevated IL-6, CCL2, and CXCL8. The vascular niche (**right**) supports CXCL12-mediated retention near sinusoidal LepR^+^ stromal cells and arteriolar NG2^+^ pericytes, with V-AML integration into the endothelial wall conferring quiescence and chemoresistance; endothelial NO drives vascular leakiness and hypoxia, while the ADM–CALCRL–RAMP2 axis preserves relapse-initiating cells.

Evidence indicates that self-renewing HSCs reside in the bone-marrow perivascular niche rather than exclusively in long-term dormant reserves (Figure 1). This specialized, protective compartment is composed of MSCs [46,47] and endothelial cells of sinusoidal microvessels [10,48]. From this location, HSCs monitor blood-borne factors and activate the recruitment of additional HSCs from quiescent endosteal niches to stimulate hematopoiesis and osteogenesis, counteracting hematological stress [49]. During embryogenesis, primitive hematopoiesis arises from hemogenic endothelium of the aorta-gonad-mesonephros region and yolk sac via endothelial-to-hematopoietic transition [50,51,52]; the close spatial relationship between endothelial and hematopoietic progenitors persists into adulthood, with the vascular niche traditionally considered comparatively oxygen-rich relative to the endosteum [53]—a view that direct intravital oxygen measurements have since challenged and partially inverted (see Section 5).

Sinusoids are fenestrated, low-pressure channels [54,55] that maintain niche homeostasis through secretion of IL-1, IL-6, IL-8, GM-CSF, TNF, G-CSF, chemokines, and matrix metalloproteinases [56]. The endosteal niche contains transition-zone vessels [57], roughly 15% of HSCs [58], and approximately 10% of marrow volume. The central vascular niche occupies the remaining ~90% of marrow volume and is estimated to harbor ~85% of HSCs [48]. The endosteal compartment is relatively drug-resistant and supports post-chemotherapy regeneration [59], whereas the vascular compartment is sensitive to irradiation and myeloablation [60]. Two-thirds of perivascular HSCs associate with sinusoids and LepR^+^ stromal cells; the remaining third associate with arterioles and NG2^+^/Nestinhigh pericytes [61,62,63].

Much of this cellular anatomy comes from mouse genetic-reporter and intravital studies, and its correspondence with human marrow is only partial. In human bone marrow, perivascular mesenchymal stromal/progenitor cells are identified most consistently by CD146 (MCAM). These CD146^+^ cells line the sinusoidal wall and support human hematopoietic stem/progenitor cells in vitro and after xenotransplantation [64]. Markers commonly used to define murine niche populations, namely Nestin, leptin receptor (LepR), NG2, and N-cadherin, are not equally validated in human marrow [65]. Cellular relationships established in mice should therefore be extrapolated to human disease with caution.

Specific alterations in the BM vascular niche can act synergistically with genetic or epigenetic lesions in hematopoietic cells to facilitate mutant-cell survival and expansion, contribute to progression and protection from chemotherapy, and promote relapse. Using intravital two-photon microscopy, Passaro et al. showed that vascular leakiness and increased hypoxia in AML are dependent on endothelial nitric oxide (NO); induction chemotherapy that fails to restore normal vasculature may be associated with poor prognosis, while inhibition of NO production promotes normal hematopoiesis and enhances treatment response in preclinical models [66]. CXCL12-abundant reticular (CAR) cells are heterogeneously distributed in the perivascular endothelium: arteriolar CARs (NG2^+^/LepR^−^) enforce HSC quiescence, whereas sinusoidal CARs (NG2^−^/LepR^+^) support retention and expansion [62,67,68]. The consistent association of HSCs with CXCL12-secreting reticular cells in both vascular and endosteal locations blurs the historical distinction between the two niches [66,69].

The leukemic vasculature is also expanded and structurally abnormal. Increased microvascular density has been documented in ALL [70], chronic myeloid leukemia (CML) [71,72,73], AML [74], and multiple myeloma (MM) [75] and carries prognostic weight [76]. Tumor neovasculature is abnormally permeable and tortuous [77,78]. Endothelial cells (ECs) and leukemic cells engage in bidirectional signaling: ECs provide trophic and paracrine support, while blasts release proangiogenic factors (FGF-2, VEGF-A, VEGF-C, angiopoietins) and cytokines such as GM-CSF [79,80]. AML cells can become quiescent and resist chemotherapy by integrating into the vascular endothelium, forming vascular tissue-associated AML (V-AML) [81,82,83]. Combining anti-angiogenic therapy with consolidation chemotherapy has been proposed as a practical approach for patients with acute leukemia [84].

Adrenomedullin (ADM) is a 52-amino-acid peptide mainly synthesized by endothelial cells, vascular smooth muscle cells, and pericytes [85,86,87]. It is expressed in many human tissues and exerts predominantly hypotensive, immunomodulatory, and vasoactive effects, including those observed during septic shock [88,89,90]. ADM signals through the calcitonin-receptor-like receptor (CALCRL) in complex with receptor activity-modifying proteins (RAMPs); among these, the CALCRL–RAMP2 heterodimer mediates canonical ADM signaling. ADM is also expressed by leukemia stem cells and defines an inflammatory signature in AML [91]. Moreover, ADM is overexpressed in solid tumors under hypoxic conditions via HIF-1 regulation [92], exhibiting pro-angiogenic activity and stimulating tumor progression and chemoresistance [93]. Our group showed that AML cell lines constitutively express ADM and its receptors to sustain proliferation and block differentiation, suppressing Cullin-family gene expression and activating PI3K/AKT and MAPK/ERK [94]. Larrue et al. subsequently demonstrated that the ADM–CALCRL axis preserves relapse-initiating cells (RICs) that persist after chemotherapy; high CALCRL or ADM expression in RICs correlates with adverse outcome, and CALCRL knockdown reduces LSC frequency and restores cytarabine sensitivity in patient-derived xenografts [95].

Events that promote blast–endothelium adhesion are of particular interest given the capacity of AML cells to integrate into the vascular wall. ADM downregulates CD38 on blasts—thereby weakening the CD38/hyaluronic-acid axis—and simultaneously upregulates CD31, enhancing trans-endothelial migration and circulatory dissemination [94,96]. In murine models, Cogle et al. documented progressive localization of AML blasts around hepatic portal vessels and their incorporation into endothelial syncytia (V-AML) that acquire quiescence and chemoresistance [83]. Analogous observations in chronic myeloproliferative neoplasms, where marrow endothelial cells carry disease-defining mutations, suggest similar endothelial participation in other hematologic malignancies.

## 5. The Osteoblastic Niche

In AML, LSCs are preferentially home to the osteoblastic compartment—the endosteal surface lined by osteoblasts—rather than to the vascular niche, as confirmed by homing and serial engraftment experiments [28,97] (Figure 1). Elevated endosteal ATP supports LSC retention through P2X7 signaling [98]. LSCs in this compartment exhibit high rates of symmetric division, strong colony-forming potential, elevated glycolytic flux, and superior leukemogenic capacity in vivo [97]. Expression of active Rac1 in murine AML reduces disease latency by enhancing niche homing, quiescence, and apoptosis protection [99].

The physiology of this compartment helps explain that preference. Oxygen tension in the leukemic endosteum falls to ~0.1% [97,100], substantially below that of the vascular niche; in healthy mice the relationship is inverted, with deeper peri-sinusoidal regions of the vascular niche slightly more hypoxic (~1.3%) than the endosteum [101].

These precise values come from murine intravital imaging; human measurements are fewer but confirm that the marrow is hypoxic relative to arterial blood [102], and the extent to which AML deepens this hypoxia in patients remains an open, therapeutically relevant question [100]. Hypoxia can promote HSC quiescence [103] and, via hypoxia-associated factor (HAF), HIF-1α, and HIF-2α, upregulates OCT-3/4, NANOG, and SOX2—transcription factors whose ectopic expression can reprogram somatic cells into induced pluripotency [104,105,106,107]. In cancer stem cells, MYC, NANOG, and SOX2 stabilize HIF-2α, which represses p53 and preserves self-renewal [108]; loss of p53 in turn impairs MDM2-mediated HIF-1α degradation [109]. Although a clear consensus has not been reached, increased expression of OCT4 and NANOG has been reported in AML relative to normal marrow [110].

In endosteal LSCs, HIF-1 drives metabolic reprogramming by upregulating PDK2 and blocking pyruvate entry into the tricarboxylic acid (TCA) cycle [111]. Concurrent AMPK activation induces GLUT1 and the pentose-phosphate pathway, raising reduced glutathione and buffering against reactive oxygen species (ROS)-mediated oxidative stress [97,112]. In T-cell acute lymphoblastic leukemia (T-ALL), NOTCH mutations engage AMPK with comparable cytoprotective effect [113], supporting AMPK as a therapeutic target for sensitization. Conversely, oxidative phosphorylation (OXPHOS) is required to maintain quiescent human LSCs [114], leaving the quantitative relationship between the endosteum, hypoxia, glucose availability, and LSC fate open.

Retention in this niche is enforced by a dense set of adhesive contacts. Blasts engage osteoblasts through fibronectin–hyaluronic-acid/CD44, ICAM1/LFA, WNT/Frizzled, Notch/Jagged, CXCL12/CXCR4, VCAM1/VLA4, and N-cadherin/N-cadherin pairings, sustaining a stemness program in vitro [115,116,117]. Osteoblasts shield AML cells from chemotherapy- and chemokine-induced apoptosis. CXCR4-high AML cells escape CXCL12-mediated apoptosis in co-culture, independently of direct contact or MSC involvement [118]; this protection is lost after histone-deacetylase inhibition through downregulation of tissue-nonspecific alkaline phosphatase [119,120]. Osteoblasts protect blasts from cytarabine in both two- and three-dimensional systems [121], and AML-derived osteoblasts shield cells from daunorubicin more effectively than healthy-donor osteoblasts [122].

The interaction is reciprocal. Osteoblast numbers are reduced in AML patients and murine models [123,124,125]. Co-culture with AML blasts arrests stromal differentiation at a pre-osteoblastic stage that overexpresses pro-leukemogenic cytokines (IL-6, CCL2, CXCL8) [126]. Elevated marrow CCL3 suppresses mature osteoblasts, as previously shown in MM [125,127]. Genetic depletion of osteoblasts accelerates leukemic engraftment, while pharmacologic preservation of osteoblasts restores marrow function, reduces tumor burden, and prolongs survival [124].

Mechanistically, blast-derived kynurenine binds serotonin receptor 1B (HTR1B) on osteoblasts, inducing serum amyloid A, which upregulates IDO1—the rate-limiting enzyme in kynurenine synthesis—and closes a self-sustaining kynurenine–HTR1B–SAA–IDO1 loop that generates a leukemia-permissive niche [128]. Secondary transplantation of long-term HSC-phenotype cells from osteoblast-ablated donors paradoxically prolongs survival, because separation from the endosteum disengages LSCs from quiescence, drives proliferation and differentiation, and depletes the LSC pool [129].

Taken together, this evidence indicates that cells expressing the characteristic LSC functional program reside in the endosteal niche through direct and indirect interactions with microenvironmental cells that recruit and anchor them there. Osteoblasts, in turn, are instructed by AML blasts either to preserve LSC properties or to actively generate functional LSCs—cells held in a state of metabolic and proliferative quiescence, resistant to oxidative stress, and shielded from chemotherapy. The osteoblastic niche therefore remains a main suspect for the persistence of measurable residual disease (MRD) during therapy.

## 6. Chemoresistance Mechanisms Induced by the Bone Marrow Microenvironment

**Figure 2:**
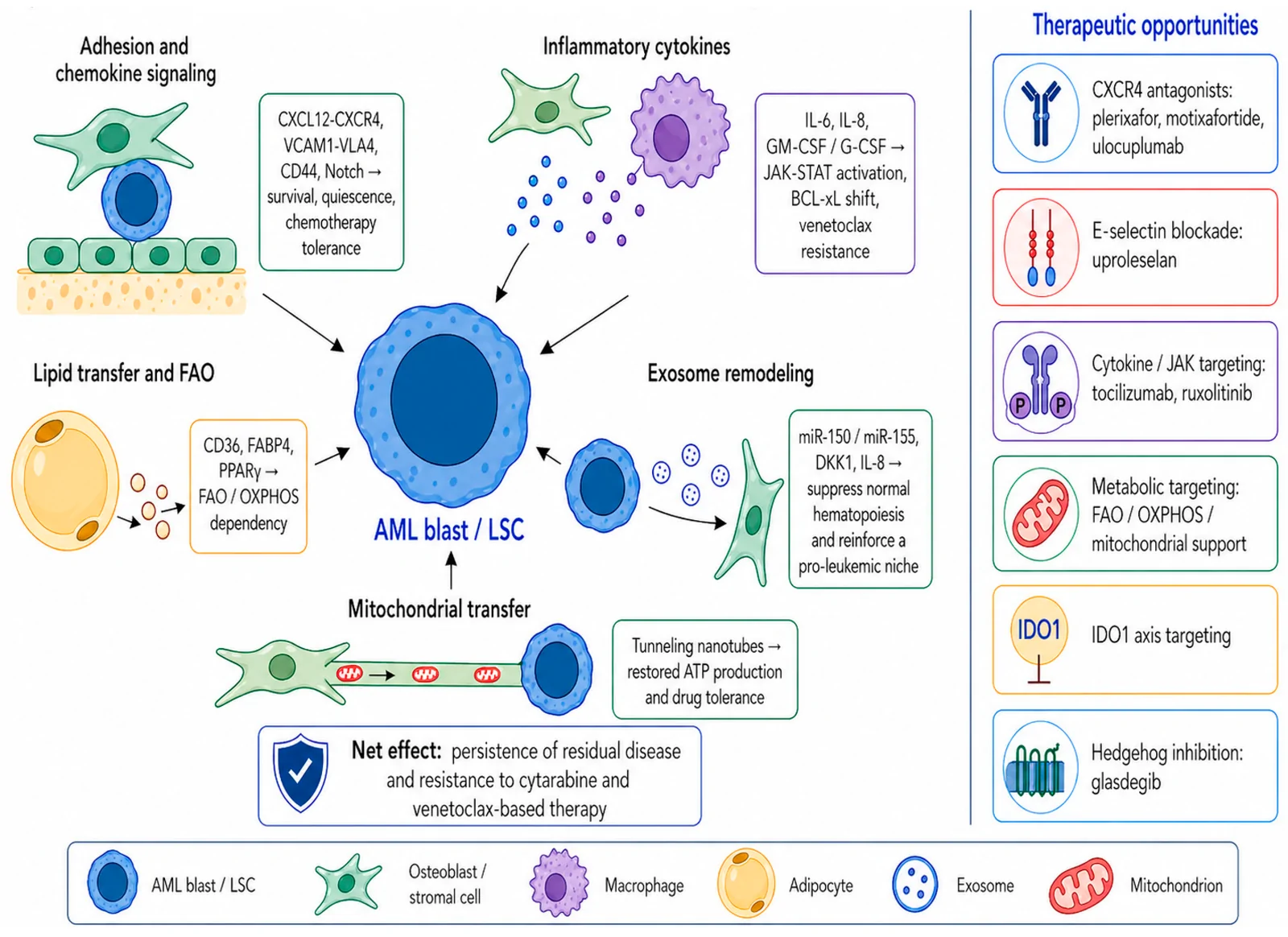
Microenvironment-driven chemoresistance and therapeutic opportunities in AML. The LSC integrates four protective inputs from the marrow microenvironment: adhesion- and chemokine-mediated survival signaling, lipid transfer from adipocytes feeding fatty-acid oxidation and BCL-2-dependent quiescence, mitochondrial transfer through tunneling nanotubes from MSCs, and stromal pro-inflammatory cytokines that engage JAK–STAT to switch anti-apoptotic dependence from BCL-2 to BCL-xL. Exosome-mediated remodeling of normal hematopoiesis (miR-150, miR-155) and stromal MSC reprogramming (IL-8) reinforce a leukemia-permissive niche. The right column lists the principal therapeutic strategies under clinical evaluation: CXCR4 antagonists (plerixafor, motixafortide, ulocuplumab), the E-selectin antagonist uproleselan, JAK and IL-6 inhibitors (ruxolitinib, tocilizumab), metabolic targeting of FAO/OXPHOS, IDO1 inhibition, and the Hedgehog inhibitor glasdegib.

Chemoresistance in AML depends on a set of non-cell-autonomous mechanisms that the preceding sections have begun to outline: MSC- and osteoblast-mediated protection, adhesion- and chemokine-driven survival signaling, immune cell-mediated drug tolerance, and metabolic reprogramming sustained by the niche (Figure 2). This section integrates those mechanisms.

Contact with MSCs from healthy donors or AML patients confers a side-population (SP) phenotype on AML blasts through α4-integrin engagement and stroma-dependent activation of ABC transporters; SP cells are quiescent and chemoresistant in vitro and in patient-derived xenografts [130]. Co-culture with AML-derived MSCs activates Notch signaling more strongly than MSCs from healthy donors, amplifying proliferation and drug tolerance [131]. Furthermore, co-culture of AML blasts with MSCs engages a self-sustaining loop in which AML cells, via the apoptosis repressor with caspase recruitment domain (ARC) protein, activate NF-κB and drive IL-1β secretion. IL-1β induces COX-2 expression in MSCs, which release prostaglandin E2 (PGE2); PGE2 feeds back onto AML cells and activates a β-catenin/ARC axis, increasing their oncogenic and chemoresistance potential [132]. ARC had previously been reported as an independent adverse prognostic factor in AML [133]. These findings are consistent with the notion that a pro-inflammatory microenvironment may be associated with greater aggressiveness, chemoresistance, and unfavorable clinical outcomes.

In the HS-5 co-culture model, stromal contact shifts AML metabolism toward OXPHOS and ATP synthesis, inhibiting AMPK and activating mTORC1 [134]. Genetic AMPK knockout accelerates leukemia growth and chemoresistance, supporting the OXPHOS–AMPK axis as a potential therapeutic target.

The CXCL12/CXCR4 axis is a major mediator of niche-mediated resistance to AML therapies, including FLT3 and IDH inhibitors [135]. CXCR4 is expressed on HSCs and is overexpressed on AML cells, where it correlates with poor prognosis; chemotherapy and the hypoxic niche further upregulate it [136,137]. High CXCR4 allows leukemic clones to outcompete normal HSCs for niche occupancy and drive their exhaustion [138]. Blast–stroma adhesion provides a protective environment from chemotherapy-induced cell death by promoting leukemic cell growth and activating anti-apoptotic signals [139]. In particular, the CXCL12/CXCR4 axis promotes leukemic cell survival by activating AKT and MAPK pathways and by overexpressing the anti-apoptotic protein BCL-xL—a consequence of CXCR4-driven downregulation of miR-let-7a, which otherwise targets BCL-xL for degradation [140]. Stromal GM-CSF, G-CSF, and other cytokines engage JAK/STAT to switch anti-apoptotic dependence from BCL-2 to BCL-xL, producing refractoriness to venetoclax that is reversed ex vivo and in murine models by ruxolitinib [141]. CXCL12 also recruits macrophages and polarizes them toward an immunosuppressive M2 phenotype [142,143,144]. Celik et al. reported that AML marrow is enriched for MSCs secreting CXCL8, which supports leukemic survival via PI3K/AKT [145].

Bone-marrow macrophages are major effectors of chemoresistance. CD169^+^ macrophages are required for cytarabine and doxorubicin resistance in murine AML [146]; M-CSF-induced CD163^+^CD206^+^ M2 macrophages protect AML cell lines from daunorubicin [147]. M2-like tumor-associated macrophages (TAMs) regulate their own polarization [148] and support leukemic cell survival by rewiring their metabolic profile to rely more on amino acids, fatty acids, and oxidative metabolism than healthy macrophages [149]. TAMs also sustain the survival of drug-resistant LSCs, which drive disease relapse [150].

Neutrophils, the most abundant myeloid cells in the marrow, also contribute to niche function. Under homeostasis, senescent neutrophils that return to the marrow are cleared by resident macrophages. This clearance follows a circadian rhythm that remodels the size and output of the hematopoietic niche and sets the timing of progenitor egress [151]. In AML, the innate immune compartment, including neutrophils, granulocytic myeloid-derived suppressor cells, and tumor-associated macrophages, is skewed toward immunosuppressive, leukemia-permissive states that blunt anti-leukemic immunity [152]. Direct mechanistic data on mature neutrophils in AML remain more limited than for macrophages, so their niche contribution, although biologically plausible, is still being defined.

LSC drug tolerance depends on metabolic plasticity shaped by the niche [153]. One important mechanism of relapse to venetoclax/azacitidine involves increased fatty-acid oxidation (FAO) in LSCs, which can be overcome experimentally by etomoxir [154,155]. FAO provides NADH, FADH_2_, and acetyl-CoA for mitochondrial respiration and the TCA cycle [156]. Bone-marrow adipocytes release CXCL12 to retain LSCs in their proximity [139] and deliver free fatty acids in response to LSC-derived TNF-α, IL-1α, IL-1β, and CSF2 [157]. LSCs upregulate the fatty-acid transporter CD36 and the lipid chaperone FABP4, and nuclear activation of PPARγ drives CD36, FABP4, and BCL2 transcription, sustaining drug resistance [158,159]. Shafat et al. confirmed that AML blasts activate adipocyte lipolysis and that FABP4 knockdown suppresses leukemic proliferation in co-culture and prolongs survival in Hoxa9/Meis1-driven AML [160]. As noted in Section 5, LSCs maintain low ROS levels; BCL-2 inhibition selectively eliminates quiescent LSCs by disrupting OXPHOS [114], and FAO inhibition triggers the integrated stress response and apoptosis [161,162,163].

Metabolic cooperation extends to organelle transfer. Following OXPHOS inhibition, AML cells form nanotubes through which functional mitochondria are transferred from stroma to leukemic cells, improving their metabolic activity and promoting drug resistance. The process is driven by ROS-mediated activation of the PI3K/AKT pathway, which opens a transmembrane gap-junction channel enabling mitochondrial trafficking; the resulting increase in mitochondrial mass and ATP production provides a survival advantage [164]. AML chemoresistance therefore reflects, at least in part, metabolic cooperation between the leukemic clone and its BMM, rather than being explained solely by cell-intrinsic mechanisms.

## 7. Remodeling of the Niche Induced by Cross-Talk with Leukemic Cells

Research has mainly described how the BMM affects leukemic cells, with only limited focus on the reciprocal relationship. Increasing evidence indicates a “partnership” between the two components, in which leukemic cells also modify the marrow niches and thereby improve the overall “fitness” of the neoplasm (Figure 1). One of the most informative mechanisms of communication between leukemic cells and their surroundings is the production of exosomes, which are principal mediators of intercellular signaling.

Exosomes carry diverse molecular cargo—RNA (especially microRNA), proteins, lipids, and DNA—and have potential as disease biomarkers or as targets for novel therapies. AML blasts use exosomes to suppress normal hematopoietic progenitors both indirectly, by altering the supporting marrow niche into a microenvironment favorable to leukemic cells (including modification of CXCL12-dependent signaling, discussed in Section 4 and Section 6), and directly, by delivering inhibitory microRNA signals to hematopoietic progenitors [165]. Exosomes isolated from leukemic blast cultures or from the plasma of AML mouse models are rich in miR-150 and miR-155, which impair the clonogenic ability of normal HSCs by inhibiting c-Myc transcription [166].

Exosomes derived from AML blasts also downregulate genes that support normal hematopoiesis in marrow stromal cells—including IGF1, CXCL12, KITL, and IL-7—as well as osteogenic genes such as IGF1, OCN, and COL1A1. At the same time, they upregulate DKK1 in the same cells, inhibiting the Wnt/β-catenin pathway, which is essential to physiological hematopoiesis and osteogenesis. Forced DKK1 expression in osteoblasts leads to osteopenia and destruction of the HSC niche, resulting in hematopoietic defects [123]. A further study demonstrated that exosomes from an AML cell line induce IL-8 production by bone marrow MSCs, which in turn promotes chemoresistance to etoposide by increasing expression of the 70 kDa heat-shock protein (HSP70) and lysosomal-associated membrane protein 3 (CD63) [167]. Overall, these studies highlight the capacity of AML cells to create a pro-leukemic niche that hinders normal hematopoiesis and activates chemotherapy-resistance mechanisms.

## 8. Therapeutic Implications

These mechanisms provide a rationale for therapeutic targeting of the BMM (Figure 2). The CXCL12/CXCR4 axis is the most extensively studied target. Plerixafor, a CXCR4 partial agonist, has been evaluated across phase I–II trials in newly diagnosed and relapsed/refractory (R/R) AML with intensive, non-intensive, salvage, and conditioning regimens [168,169,170,171,172,173,174,175,176]. In responders it at least doubles blast mobilization into peripheral blood [169,170]. Trial NCT00906945 was terminated for futility at interim analysis, and historical comparisons have shown no consistent survival advantage [175]. No randomized data are available.

Motixafortide (BL-8040), LY2510924, and the IgG4 antibody ulocuplumab have produced encouraging activity in R/R AML [177,178]. Ulocuplumab—exploiting IgG pharmacokinetics and potential antibody-dependent cellular cytotoxicity—administered one week before mitoxantrone–etoposide–cytarabine (MEC) achieved fivefold blast mobilization, four complete remissions (CR) or CR with incomplete count recovery (CRi) before chemotherapy, and an overall response rate (ORR) of 51% [179]. Nonetheless, randomized trials of motixafortide plus high-dose cytarabine (HiDAC) consolidation and of ulocuplumab plus low-dose cytarabine (LDAC) in unfit patients failed to improve response or overall survival (OS) [180,181]. Timing and intensity of chemotherapy are therefore likely determinants of synergy.

Tipifarnib, a farnesyltransferase inhibitor extensively studied in AML [182,183,184,185,186], illustrates the same principle. Re-analysis of NCT00027872 identified high baseline CXCL12 expression as predictive of response, a finding replicated in R/R peripheral T-cell lymphoma [187,188,189]; tipifarnib reduces CXCL12 transcription in pancreatic and bone-marrow stromal cells in vitro and ex vivo [188,190]. Yet phase III trials in newly diagnosed elderly unfit patients and as post-induction maintenance showed no response or survival benefit [191,192,193]. Early-phase combinations with chemotherapy produced prolonged CR duration in matched historical comparisons but have not been validated in randomized studies [194,195,196,197].

Dociparstat (CX-01), a 2-O,3-O-desulfated heparin without anticoagulant activity, antagonizes CXCL12, PF4, HMGB1, and P-selectin [198,199]. Disruption of the CXCL12 signal is associated not with mobilization of AML blasts but with inhibition of chemotaxis [198,200]. In combination with 7 + 3 induction it delivered CR/CRi rates approaching 90% versus 50% for chemotherapy alone, with longer event-free survival (EFS); the randomized phase III NCT04571645 was terminated for slow accrual [198,201,202]. Its pleiotropic effects on PF4 and P-selectin may additionally mitigate chemotherapy-induced cytopenias and blast adhesion [199,203].

Uproleselan (GMI-1271), a synthetic glycomimetic E-selectin antagonist, targets cell adhesion to the endothelium and the pro-survival signaling it activates [204,205]. Early-phase trials reported ORRs of 48%, 80%, and 39% with salvage, induction, and bridge-to-transplant regimens in cohorts enriched for adverse cytogenetics and secondary AML [206,207]. The phase III trial combining uproleselan with MEC or FLAI did not meet its OS primary endpoint (hazard ratio, HR, 0.89; 95% CI 0.69–1.15) but suggested a significant benefit in refractory disease (31.2 vs. 10.1 months) [208], consistent with higher E-selectin ligand expression on refractory blasts [206]. CR/CRi rates with motixafortide have similarly been higher in refractory than relapsed disease [169,178], suggesting that chemorefractory patients may preferentially benefit from niche-targeted therapy.

β-catenin dysregulation can be disrupted by sequestering the coactivator CBP with the peptidomimetic CWP232291 [209], by inhibiting the MUC1-C/β-catenin interaction with GO-203 [210,211,212], or by GSK-3 inhibition. As single agents in R/R AML, all three failed to produce meaningful response despite acceptable safety; only GO-203 combined with decitabine achieved a 43% CR/CRi rate [213,214,215]. The kynurenine–HTR1B–SAA–IDO1 axis has been addressed through IDO1 inhibition: epacadostat development was halted, while indoximod with standard induction delivered 84% CR/CRi and MRD negativity in half of a small, predominantly adverse-risk cohort [216,217,218]. Bioinformatic analyses link IDO1 overexpression to altered B-cell subsets and elevated IL-10, both reversible in vitro by IDO1 inhibition [219].

Pro-inflammatory cytokines—IL-1β, IL-6, IL-8—released by blasts, MSCs, and pre-osteoblasts sustain the altered niche and drive resistance to daunorubicin and cytarabine, with IL-6 as a recurrent mediator [220,221]. In the phase I TOCILAM trial, tocilizumab added to standard induction produced an acceptable response, but one-year survival was below 50%, consistent with the adverse/intermediate European LeukemiaNet (ELN) 2017 risk profile [222].

The Hedgehog pathway regulates HSC quiescence and LSC maintenance [223,224,225]. AML cells exploit both autocrine PTCH1 signaling and paracrine Hh stimulation from osteoblasts, endothelial cells, and stroma. Smoothened inhibitors have shown limited activity; glasdegib as a single agent is active only in R/R AML [226,227,228]. In newly diagnosed patients, randomized trials demonstrated CR/CRi and OS benefit for glasdegib plus LDAC but only a CR/CRi benefit when combined with hypomethylating agents [229,230,231,232,233]. The randomized phase III BRIGHT AML 1019 did not confirm an OS advantage (HR 1.05; 95% CI 0.782–1.408; *p* = 0.749) despite earlier evidence of cytarabine sensitization through UGT1A downregulation [231,234,235]; placebo was favored in ELN 2017 intermediate-risk and GLI2-mutated subgroups.

Overall, with the exception of glasdegib plus LDAC, no niche-directed strategy has entered standard practice. Activity of single-axis inhibition remains limited, largely because of the complexity and redundancy of BMM signaling networks, so combination strategies are being explored. Focal adhesion kinase (FAK) is a promising target, and its pharmacological blockade has been shown to synergize with either anti-CD44 antibody or BCL-2 inhibitors in preclinical models [236,237,238].

The increasing availability of different bispecific and trispecific monoclonal antibody platforms may also enable multitarget strategies within a single molecule. T-cell redirection currently represents the most extensively developed modality. In a recent study, an anti-IL1RL1 × anti-CD3 T-cell engager, significantly improved survival in xenograft models by exploiting the AML-supportive IL-33/IL1RL1 axis, which may involve both stromal-derived and autocrine LSC-derived IL-33 signals [239].

Microenvironment-directed treatments timing and subgroup selection, informed by niche and disease biology (secondary, therapy-related, refractory, and high-risk disease), are likely to be important determinants of clinical activity and will require rigorous definition in future trials.

## 9. Conclusions

The BMM has a major role in AML biology and pathogenesis, sustaining leukemic cell survival, proliferation, and drug resistance through a network of cellular and molecular interactions. The vascular and osteoblastic niches, together with adipocytes and tumor-associated macrophages, are remodeled by AML blasts into a permissive compartment that favors disease progression. Metabolic reprogramming—including enhanced fatty-acid oxidation and mitochondrial transfer from stromal cells—contributes to chemoresistance, while exosome-mediated communication allows AML cells to further reshape the niche, suppress normal hematopoiesis, and reinforce leukemic fitness.

The tumor microenvironment, although composed of non-neoplastic cells, must therefore be considered in any comprehensive model of AML biology. In selected contexts—stromal-initiated murine models, donor cell leukemia, and DCL after cord-blood transplantation—it may also contribute to leukemogenesis.

For therapy, the implication is not to replace clone-directed treatment but to combine it with strategies that disrupt protective niche interactions, quiescence, and metabolic plasticity. Preliminary clinical signals with CXCR4, E-selectin, IDO1, and Hedgehog-directed approaches are encouraging in some settings, particularly refractory, secondary, or therapy-related AML, but randomized confirmation, rational combinations, and better patient selection remain necessary.

## Data Availability

No new data were created or analyzed in this study. Data sharing is not applicable to this article.

## References

[1] Darwin C. (1861). On the Origin of Species.

[2] Till J.E., Mcculloch E.A., Siminovitch L. (1964). A stochastic model of stem cell proliferation, based on the growth of spleen colony-forming cells. Proc. Natl. Acad. Sci. USA.

[3] Reya T., Morrison S.J., Clarke M.F., Weissman I.L. (2001). Stem cells, cancer, and cancer stem cells. Nature.

[4] Lapidot T., Sirard C., Vormoor J., Murdoch B., Hoang T., Caceres-Cortes J., Minden M., Paterson B., Caligiuri M.A., Dick J.E. (1994). A cell initiating human acute myeloid leukaemia after transplantation into SCID mice. Nature.

[5] Tan B.T., Park C.Y., Ailles L.E., Weissman I.L. (2006). The cancer stem cell hypothesis: A work in progress. Lab Investig. J. Tech Methods Pathol..

[6] Shultz L.D., Ishikawa F., Greiner D.L. (2007). Humanized mice in translational biomedical research. Nat. Rev. Immunol..

[7] Sarry J.E., Murphy K., Perry R., Sanchez P.V., Secreto A., Keefer C., Swider C.R., Strzelecki A.-C., Cavelier C., Récher C. (2011). Human acute myelogenous leukemia stem cells are rare and heterogeneous when assayed in NOD/SCID/IL2Rγc-deficient mice. J. Clin. Investig..

[8] Junttila M.R., de Sauvage F.J. (2013). Influence of tumour micro-environment heterogeneity on therapeutic response. Nature.

[9] Pinho S., Frenette P.S. (2019). Haematopoietic stem cell activity and interactions with the niche. Nat. Rev. Mol. Cell Biol..

[10] Morrison S.J., Scadden D.T. (2014). The bone marrow niche for haematopoietic stem cells. Nature.

[11] Ladikou E.E., Sivaloganathan H., Pepper A., Chevassut T. (2020). Acute Myeloid Leukaemia in Its Niche: The Bone Marrow Microenvironment in Acute Myeloid Leukaemia. Curr. Oncol. Rep..

[12] Rashidi A., Uy G.L. (2015). Targeting the Microenvironment in Acute Myeloid Leukemia. Curr. Hematol. Malig. Rep..

[13] Wang A., Zhong H. (2018). Roles of the bone marrow niche in hematopoiesis, leukemogenesis, and chemotherapy resistance in acute myeloid leukemia. Hematology.

[14] Kolosova A., Mould I., Pepper C., Mitchell S., Pepper A., Ladikou E., Simoes F. (2026). Exploring the Bone Marrow Microenvironment as a Therapeutic Barrier and Targetable Source of Crosstalk in Acute Myeloid Leukemia. Blood Lymphat. Cancer.

[15] Barrett A.J. (2008). Understanding and harnessing the graft-versus-leukaemia effect. Br. J. Haematol..

[16] Barrett A.J., Battiwalla M. (2010). Relapse after allogeneic stem cell transplantation. Expert Rev. Hematol..

[17] Kato M., Yamashita T., Suzuki R., Matsumoto K., Nishimori H., Takahashi S., Iwato K., Nakaseko C., Kondo T., Imada K. (2016). Donor cell-derived hematological malignancy: A survey by the Japan Society for Hematopoietic Cell Transplantation. Leukemia.

[18] Engel N., Rovo A., Badoglio M., Labopin M., Basak G.W., Beguin Y., Guyotat D., Ljungman P., Nagler A., Schattenberg A. (2019). European experience and risk factor analysis of donor cell-derived leukaemias/MDS following haematopoietic cell transplantation. Leukemia.

[19] Yanada M., Konuma T., Yamasaki S., Kondo T., Fukuda T., Shingai N., Sawa M., Ozawa Y., Tanaka M., Uchida N. (2021). Relapse of acute myeloid leukemia after allogeneic hematopoietic cell transplantation: Clinical features and outcomes. Bone Marrow Transplant..

[20] Williams L., Doucette K., Karp J.E., Lai C. (2021). Genetics of donor cell leukemia in acute myelogenous leukemia and myelodysplastic syndrome. Bone Marrow Transpl..

[21] Yasuda T., Ueno T., Fukumura K., Yamato A., Ando M., Yamaguchi H., Soda M., Kawazu M., Sai E., Yamashita Y. (2014). Leukemic evolution of donor-derived cells harboring IDH2 and DNMT3A mutations after allogeneic stem cell transplantation. Leukemia.

[22] Gondek L.P., Zheng G., Ghiaur G., DeZern A.E., Matsui W., Yegnasubramanian S., Lin M.-T., Levis M., Eshleman J.R., Varadhan R. (2016). Donor cell leukemia arising from clonal hematopoiesis after bone marrow transplantation. Leukemia.

[23] Frick M., Chan W., Arends C.M., Hablesreiter R., Halik A., Heuser M., Michonneau D., Blau O., Hoyer K., Christen F. (2019). Role of Donor Clonal Hematopoiesis in Allogeneic Hematopoietic Stem-Cell Transplantation. J. Clin. Oncol. Off. J. Am. Soc. Clin. Oncol..

[24] Duarte D., Hawkins E.D., Lo Celso C. (2018). The interplay of leukemia cells and the bone marrow microenvironment. Blood.

[25] Witkowski M.T., Kousteni S., Aifantis I. (2020). Mapping and targeting of the leukemic microenvironment. J. Exp. Med..

[26] Raaijmakers M.H.G.P., Mukherjee S., Guo S., Zhang S., Kobayashi T., Schoonmaker J.A., Ebert B.L., Al-Shahrour F., Hasserjian R.P., Scadden E.O. (2010). Bone progenitor dysfunction induces myelodysplasia and secondary leukaemia. Nature.

[27] Colmone A., Amorim M., Pontier A.L., Wang S., Jablonski E., Sipkins D.A. (2008). Leukemic cells create bone marrow niches that disrupt the behavior of normal hematopoietic progenitor cells. Science.

[28] Ishikawa F., Yoshida S., Saito Y., Hijikata A., Kitamura H., Tanaka S., Nakamura R., Tanaka T., Tomiyama H., Saito N. (2007). Chemotherapy-resistant human AML stem cells home to and engraft within the bone-marrow endosteal region. Nat. Biotechnol..

[29] Aldoss I., Clark M., Marcucci G., Forman S.J. (2021). Donor derived leukemia in allogeneic transplantation. Leuk. Lymphoma.

[30] Aldoss I., Song J.Y., Curtin P.T., Forman S.J. (2020). Multiple donor-derived leukemias in a recipient of allogeneic hematopoietic cell transplantation for myeloid malignancy. Blood Adv..

[31] Goulard M., Dosquet C., Bonnet D. (2018). Role of the microenvironment in myeloid malignancies. Cell. Mol. Life Sci. CMLS.

[32] Geyh S., Oz S., Cadeddu R.P., Fröbel J., Brückner B., Kündgen A., Fenk R., Bruns I., Zilkens C., Hermsen D. (2013). Insufficient stromal support in MDS results from molecular and functional deficits of mesenchymal stromal cells. Leukemia.

[33] Ferrer R.A., Wobus M., List C., Wehner R., Schönefeldt C., Brocard B., Mohr B., Rauner M., Schmitz M., Stiehler M. (2013). Mesenchymal stromal cells from patients with myelodyplastic syndrome display distinct functional alterations that are modulated by lenalidomide. Haematologica.

[34] Geyh S., Rodríguez-Paredes M., Jäger P., Koch A., Bormann F., Gutekunst J., Zilkens C., Germing U., Kobbe G., Lyko F. (2018). Transforming growth factor β1-mediated functional inhibition of mesenchymal stromal cells in myelodysplastic syndromes and acute myeloid leukemia. Haematologica.

[35] Santamaría C., Muntión S., Rosón B., Blanco B., López-Villar O., Carrancio S., Sanchez-Guijo F.M., Diez-Campelo M., Alvarez-Fernandez S., Sarasquete M.E. (2012). Impaired expression of DICER, DROSHA, SBDS and some microRNAs in mesenchymal stromal cells from myelodysplastic syndrome patients. Haematologica.

[36] Ozdogan H., Gur Dedeoglu B., Oztemur Islakoglu Y., Aydos A., Kose S., Atalay A., Yegin Z.A., Avcu F., Uckan Cetinkaya D., Ilhan O. (2017). DICER1 gene and miRNA dysregulation in mesenchymal stem cells of patients with myelodysplastic syndrome and acute myeloblastic leukemia. Leuk. Res..

[37] Zhao Y., Wu D., Fei C., Guo J., Gu S., Zhu Y., Xu F., Zhang Z., Wu L., Li X. (2015). Down-regulation of Dicer1 promotes cellular senescence and decreases the differentiation and stem cell-supporting capacities of mesenchymal stromal cells in patients with myelodysplastic syndrome. Haematologica.

[38] Kode A., Manavalan J.S., Mosialou I., Bhagat G., Rathinam C.V., Luo N., Khiabanian H., Lee A., Murty V.V., Friedman R. (2014). Leukaemogenesis induced by an activating β-catenin mutation in osteoblasts. Nature.

[39] Kode A., Mosialou I., Manavalan S.J., Rathinam C.V., Friedman R.A., Teruya-Feldstein J., Bhagat G., Berman E., Kousteni S. (2016). FoxO1-dependent induction of acute myeloid leukemia by osteoblasts in mice. Leukemia.

[40] Bhagat T.D., Chen S., Bartenstein M., Barlowe A.T., Von Ahrens D., Choudhary G.S., Tivnan P., Amin E., Marcondes A.M., Sanders M.A. (2017). Epigenetically Aberrant Stroma in MDS Propagates Disease via Wnt/β-Catenin Activation. Cancer Res..

[41] Hu Y., Chen Y., Douglas L., Li S. (2009). Beta-Catenin is essential for survival of leukemic stem cells insensitive to kinase inhibition in mice with BCR-ABL-induced chronic myeloid leukemia. Leukemia.

[42] Luis T.C., Naber B.A.E., Roozen P.P.C., Brugman M.H., de Haas E.F.E., Ghazvini M., Fibbe W.E., van Dongen J.J., Fodde R., Staal F.J. (2011). Canonical wnt signaling regulates hematopoiesis in a dosage-dependent fashion. Cell Stem Cell.

[43] Stoddart A., Wang J., Fernald A.A., Davis E.M., Johnson C.R., Hu C., Cheng J.X., McNerney M.E., Le Beau M.M. (2020). Cytotoxic Therapy-Induced Effects on Both Hematopoietic and Marrow Stromal Cells Promotes Therapy-Related Myeloid Neoplasms. Blood Cancer Discov..

[44] Kutyna M.M., Kok C.H., Lim Y., Tran E.N.H., Campbell D., Paton S., Thompson-Peach C., Lim K., Cakouros D., Arthur A. (2022). A senescence stress secretome is a hallmark of therapy-related myeloid neoplasm stromal tissue occurring soon after cytotoxic exposure. Leukemia.

[45] Özdemir C., Muratoğlu B., Özel B.N., Alpdündar-Bulut E., Tonyalı G., Ünal Ş., Uçkan-Çetinkaya D. (2023). Multiparametric analysis of etoposide exposed mesenchymal stem cells and Fanconi anemia cells: Implications in development of secondary myeloid malignancy. Clin. Exp. Med..

[46] Wang Y.X., Deng Z.H., Li Y.Y., Bai K., Ma J., Liu Y., Chen Q. (2025). Function of hematopoiesis and bone marrow niche in inflammation and non-hematopoietic diseases. Life Med..

[47] Szade K., Gulati G.S., Chan C.K.F., Kao K.S., Miyanishi M., Marjon K.D., Sinha R., George B.M., Chen J.Y., Weissman I.L. (2018). Where Hematopoietic Stem Cells Live: The Bone Marrow Niche. Antioxid. Redox Signal.

[48] Acar M., Kocherlakota K.S., Murphy M.M., Peyer J.G., Oguro H., Inra C.N., Jaiyeola C., Zhao Z., Luby-Phelps K., Morrison S.J. (2015). Deep imaging of bone marrow shows non-dividing stem cells are mainly perisinusoidal. Nature.

[49] Wilson A., Laurenti E., Oser G., van der Wath R.C., Blanco-Bose W., Jaworski M., Offner S., Dunant C.F., Eshkind L., Bockamp E. (2008). Hematopoietic Stem Cells Reversibly Switch from Dormancy to Self-Renewal during Homeostasis and Repair. Cell.

[50] Bertrand J.Y., Giroux S., Golub R., Klaine M., Jalil A., Boucontet L., Godin I., Cumano A. (2005). Characterization of purified intraembryonic hematopoietic stem cells as a tool to define their site of origin. Proc. Natl. Acad. Sci. USA.

[51] Lancrin C., Sroczynska P., Stephenson C., Allen T., Kouskoff V., Lacaud G. (2009). The haemangioblast generates haematopoietic cells through a haemogenic endothelium stage. Nature.

[52] Kissa K., Herbomel P. (2010). Blood stem cells emerge from aortic endothelium by a novel type of cell transition. Nature.

[53] Kunisaki Y., Frenette P.S. (2014). Influences of vascular niches on hematopoietic stem cell fate. Int. J. Hematol..

[54] Orr F.W., Wang H.H., Lafrenie R.M., Scherbarth S., Nance D.M. (2000). Interactions between cancer cells and the endothelium in metastasis. J. Pathol..

[55] Nombela-Arrieta C., Manz M.G. (2017). Quantification and three-dimensional microanatomical organization of the bone marrow. Blood Adv..

[56] Kobayashi H., Butler J.M., O’Donnell R., Kobayashi M., Ding B.S., Bonner B., Chiu V.K., Nolan D.J., Shido K., Benjamin L. (2010). Angiocrine factors from Akt-activated endothelial cells balance self-renewal and differentiation of haematopoietic stem cells. Nat. Cell Biol..

[57] Bixel M.G., Kusumbe A.P., Ramasamy S.K., Sivaraj K.K., Butz S., Vestweber D., Adams R.H. (2017). Flow Dynamics and HSPC Homing in Bone Marrow Microvessels. Cell Rep..

[58] Beerman I., Luis T.C., Singbrant S., Lo Celso C., Méndez-Ferrer S. (2017). The evolving view of the hematopoietic stem cell niche. Exp. Hematol..

[59] Zhao M., Tao F., Venkatraman A., Li Z., Smith S.E., Unruh J., Chen S., Ward C., Qian P., Perry J.M. (2019). N-Cadherin-Expressing Bone and Marrow Stromal Progenitor Cells Maintain Reserve Hematopoietic Stem Cells. Cell Rep..

[60] Hooper A.T., Butler J.M., Nolan D.J., Kranz A., Iida K., Kobayashi M., Kopp H.-G., Shido K., Petit I., Yanger K. (2009). Engraftment and Reconstitution of Hematopoiesis Is Dependent on VEGFR2-Mediated Regeneration of Sinusoidal Endothelial Cells. Cell Stem Cell.

[61] Asada N., Takeishi S., Frenette P.S. (2017). Complexity of bone marrow hematopoietic stem cell niche. Int. J. Hematol..

[62] Kunisaki Y., Bruns I., Scheiermann C., Ahmed J., Pinho S., Zhang D., Mizoguchi T., Wei Q., Lucas D., Ito K. (2013). Arteriolar niches maintain haematopoietic stem cell quiescence. Nature.

[63] Silberstein L.E., Lin C.P. (2013). A New Image of the Hematopoietic Stem Cell Vascular Niche. Cell Stem Cell.

[64] Corselli M., Chin C.J., Parekh C., Sahaghian A., Wang W., Ge S., Evseenko D., Wang X., Montelatici E., Lazzari L. (2013). Perivascular support of human hematopoietic stem/progenitor cells. Blood.

[65] Sá da Bandeira D., Casamitjana J., Crisan M. (2017). Pericytes, integral components of adult hematopoietic stem cell niches. Pharmacol. Ther..

[66] Passaro D., Di Tullio A., Abarrategi A., Rouault-Pierre K., Foster K., Ariza-McNaughton L., Montaner B., Chakravarty P., Bhaw L., Diana G. (2017). Increased Vascular Permeability in the Bone Marrow Microenvironment Contributes to Disease Progression and Drug Response in Acute Myeloid Leukemia. Cancer Cell.

[67] Mendelson A., Frenette P.S. (2014). Hematopoietic stem cell niche maintenance during homeostasis and regeneration. Nat. Med..

[68] Sugiyama T., Kohara H., Noda M., Nagasawa T. (2006). Maintenance of the Hematopoietic Stem Cell Pool by CXCL12-CXCR4 Chemokine Signaling in Bone Marrow Stromal Cell Niches. Immunity.

[69] Cheng H., Sun G., Cheng T. (2018). Hematopoiesis and microenvironment in hematological malignancies. Cell Regen..

[70] Dander E., Palmi C., D’Amico G., Cazzaniga G. (2021). The Bone Marrow Niche in B-Cell Acute Lymphoblastic Leukemia: The Role of Microenvironment from Pre-Leukemia to Overt Leukemia. Int. J. Mol. Sci..

[71] Aguayo A., Kantarjian H., Manshouri T., Gidel C., Estey E., Thomas D., Koller C., Estrov Z., O’Brien S., Keating M. (2000). Angiogenesis in acute and chronic leukemias and myelodysplastic syndromes. Blood.

[72] Lundberg L.G., Lerner R., Sundelin P., Rogers R., Folkman J., Palmblad J. (2000). Bone Marrow in Polycythemia Vera, Chronic Myelocytic Leukemia, and Myelofibrosis Has an Increased Vascularity. Am. J. Pathol..

[73] Korkolopoulou P., Viniou N., Kavantzas N., Patsouris E., Thymara I., Pavlopoulos P.M., Terpos E., Stamatopoulos K., Plata E., Anargyrou K. (2003). Clinicopathologic correlations of bone marrow angiogenesis in chronic myeloid leukemia: A morphometric study. Leukemia.

[74] Hussong J.W., Rodgers G.M., Shami P.J. (2000). Evidence of increased angiogenesis in patients with acute myeloid leukemia. Blood.

[75] Giuliani N., Storti P., Bolzoni M., Palma B.D., Bonomini S. (2011). Angiogenesis and Multiple Myeloma. Cancer Microenviron..

[76] Nico B., Benagiano V., Mangieri D., Maruotti N., Vacca A., Ribatti D. (2008). Evaluation of microvascular density in tumors: Pro and contra. Histol. Histopathol..

[77] Shahrabi S., Rezaeeyan H., Ahmadzadeh A., Shahjahani M., Saki N. (2016). Bone Marrow Blood Vessels: Normal and Neoplastic Niche. Oncol. Rev..

[78] Katayama Y., Uchino J., Chihara Y., Tamiya N., Kaneko Y., Yamada T., Takayama K. (2019). Tumor Neovascularization and Developments in Therapeutics. Cancers.

[79] Hassanshahi M., Hassanshahi A., Khabbazi S., Su Y.W., Xian C.J. (2017). Bone marrow sinusoidal endothelium: Damage and potential regeneration following cancer radiotherapy or chemotherapy. Angiogenesis.

[80] Lugano R., Ramachandran M., Dimberg A. (2020). Tumor angiogenesis: Causes, consequences, challenges and opportunities. Cell Mol. Life Sci..

[81] Cogle C.R., Bosse R.C., Brewer T., Migdady Y., Shirzad R., Kampen K.R., Saki N. (2016). Acute myeloid leukemia in the vascular niche. Cancer Lett..

[82] Cogle C.R., Madlambayan G.J., Goldman D.C., Al Masri A., Leon R.P., Dunlap J., Kovacsovics T., Liu Q., Meacham A., Scott E.W. (2011). Acute Myeloid Leukemia Cells Generate Leukemic Endothelial Cells with Leukemogenic Potential: Blood Vessels As Sanctuaries for Leukemia Relapse. Blood.

[83] Cogle C.R., Goldman D.C., Madlambayan G.J., Leon R.P., Al Masri A., Clark H.A., Asbaghi S.A., Tyner J.W., Dunlap J., Fan G. (2014). Functional integration of acute myeloid leukemia into the vascular niche. Leukemia.

[84] Ma J., Waxman D.J. (2008). Combination of antiangiogenesis with chemotherapy for more effective cancer treatment. Mol. Cancer Ther..

[85] Berenguer-Daizé C., Boudouresque F., Bastide C., Tounsi A., Benyahia Z., Acunzo J., Dussault N., Delfino C., Baeza N., Daniel L. (2013). Adrenomedullin Blockade Suppresses Growth of Human Hormone–Independent Prostate Tumor Xenograft in Mice. Clin. Cancer Res..

[86] Sugo S., Minamino N., Shoji H., Kangawa K., Kitamura K., Eto T., Matsuo H. (1994). Production and Secretion of Adrenomedullin from Vascular Smooth-Muscle Cells: Augmented Production by Tumor Necrosis Factor-α. Biochem. Biophys. Res. Commun..

[87] Karpinich N.O., Hoopes S.L., Kechele D.O., Lenhart P.M., Caron K.M. (2011). Adrenomedullin Function in Vascular Endothelial Cells: Insights from Genetic Mouse Models. Curr. Hypertens. Rev..

[88] Nikitenko L.L., Smith D.M., Hague S., Wilson C.R., Bicknell R., Rees M.C.P. (2002). Adrenomedullin and the microvasculature. Trends Pharmacol. Sci..

[89] Rullé S., Kioon M.D.A., Asensio C., Mussard J., Ea H.K., Boissier M.C., Lioté F., Falgarone G. (2012). Adrenomedullin, a neuropeptide with immunoregulatory properties induces semi-mature tolerogenic dendritic cells. Immunology.

[90] Kitamura K., Kangawa K., Kawamoto M., Ichiki Y., Nakamura S., Matsuo H., Eto T. (1993). Adrenomedullin: A Novel Hypotensive Peptide Isolated from Human Pheochromocytoma. Biochem Biophys. Res. Commun..

[91] Simonetti G., Angeli D., Petracci E., Fonzi E., Vedovato S., Sperotto A., Padella A., Ghetti M., Ferrari A., Robustelli V. (2021). Adrenomedullin Expression Characterizes Leukemia Stem Cells and Associates With an Inflammatory Signature in Acute Myeloid Leukemia. Front. Oncol..

[92] Nguyen S.V., Claycomb W.C. (1999). Hypoxia Regulates the Expression of the Adrenomedullin and HIF-1 Genes in Cultured HL-1 Cardiomyocytes. Biochem. Biophys. Res. Commun..

[93] Nakamura M., Han B., Nunobiki O., Kakudo K. (2006). Adrenomedullin: A Tumor Progression Factor via Angiogenic Control. Curr. Cancer Drug Targets.

[94] Di Liddo R., Bridi D., Gottardi M., De Angeli S., Grandi C., Tasso A., Bertalot T., Martinelli G., Gherlinzoni F., Conconi M.T. (2016). Adrenomedullin in the growth modulation and differentiation of acute myeloid leukemia cells. Int. J. Oncol..

[95] Larrue C., Guiraud N., Mouchel P.L., Dubois M., Farge T., Gotanègre M., Bosc C., Saland E., Nicolau-Travers M.-L., Sabatier M. (2021). Adrenomedullin-CALCRL axis controls relapse-initiating drug tolerant acute myeloid leukemia cells. Nat. Commun..

[96] Gallay N., Anani L., Lopez A., Colombat P., Binet C., Domenech J., Weksler B.B., Malavasi F., Herault O. (2007). The Role of Platelet/Endothelial Cell Adhesion Molecule–1 (CD31) and CD38 Antigens in Marrow Microenvironmental Retention of Acute Myelogenous Leukemia Cells. Cancer Res..

[97] Hao X., Gu H., Chen C., Huang D., Zhao Y., Xie L., Zou Y., Shu H.S., Zhang Y., He X. (2019). Metabolic Imaging Reveals a Unique Preference of Symmetric Cell Division and Homing of Leukemia-Initiating Cells in an Endosteal Niche. Cell Metab..

[98] He X., Wan J., Yang X., Zhang X., Huang D., Li X., Zou Y., Chen C., Yu Z., Xie L. (2021). Bone marrow niche ATP levels determine leukemia-initiating cell activity via P2X7 in leukemic models. J. Clin. Investig..

[99] Chen S., Li H., Li S., Yu J., Wang M., Xing H., Tang K., Tian Z., Rao Q., Wang J. (2016). Rac1 GTPase Promotes Interaction of Hematopoietic Stem/Progenitor Cell with Niche and Participates in Leukemia Initiation and Maintenance in Mouse. Stem Cells.

[100] Rieger C.T., Fiegl M. (2016). Microenvironmental oxygen partial pressure in acute myeloid leukemia: Is there really a role for hypoxia?. Exp. Hematol..

[101] Spencer J.A., Ferraro F., Roussakis E., Klein A., Wu J., Runnels J.M., Zaher W., Mortensen L.J., Alt C., Turcotte R. (2014). Direct measurement of local oxygen concentration in the bone marrow of live animals. Nature.

[102] Harrison J.S., Rameshwar P., Chang V., Bandari P. (2002). Oxygen saturation in the bone marrow of healthy volunteers. Blood.

[103] Guitart A.V., Hammoud M., Dello Sbarba P., Ivanovic Z., Praloran V. (2010). Slow-cycling/quiescence balance of hematopoietic stem cells is related to physiological gradient of oxygen. Exp. Hematol..

[104] Koh M.Y., Lemos R., Liu X., Powis G. (2011). The Hypoxia-Associated Factor Switches Cells from HIF-1α- to HIF-2α-Dependent Signaling Promoting Stem Cell Characteristics, Aggressive Tumor Growth and Invasion. Cancer Res..

[105] Das B., Pal B., Bhuyan R., Li H., Sarma A., Gayan S., Talukdar J., Sandhya S., Bhuyan S., Gogoi G. (2019). MYC Regulates the HIF2α Stemness Pathway via Nanog and Sox2 to Maintain Self-Renewal in Cancer Stem Cells versus Non-Stem Cancer Cells. Cancer Res..

[106] Huang J., Zhang Y., Bersenev A., O’Brien W.T., Tong W., Emerson S.G., Klein P.S. (2009). Pivotal role for glycogen synthase kinase-3 in hematopoietic stem cell homeostasis in mice. J. Clin. Investig..

[107] Zhao Q., Ren H., Feng S., Chi Y., He Y., Yang D., Ma F., Li J., Lu S., Chen F. (2015). Aberrant expression and significance of OCT-4A transcription factor in leukemia cells. Blood Cells Mol. Dis..

[108] Yi J., Zhou L.Y., Yi Y.Y., Zhu X., Su X.Y., Zhao Q., Lin J., Qian J., Deng Z.-Q. (2019). Low Expression of Pseudogene POU5F1B Affects Diagnosis and Prognosis in Acute Myeloid Leukemia (AML). Med. Sci. Monit. Int. Med. J. Exp. Clin. Res..

[109] Aanei C.M., Devêvre E., Șerban A., Tavernier-Tardy E., Guyotat D., Campos Catafal L. (2023). High-Dimensional Mass Cytometry Analysis of Embryonic Antigens and Their Signaling Pathways in Myeloid Cells from Bone Marrow Aspirates in AML Patients at Diagnosis. Cancers.

[110] Dello Sbarba P., Cheloni G. (2019). Tissue ‘Hypoxia’ and the Maintenance of Leukemia Stem Cells. Adv. Exp. Med. Biol..

[111] Wang Y., Liu Y., Malek S.N., Zheng P., Liu Y. (2011). Targeting HIF1α eliminates cancer stem cells in hematological malignancies. Cell Stem Cell.

[112] Saito Y., Chapple R.H., Lin A., Kitano A., Nakada D. (2015). AMPK Protects Leukemia-Initiating Cells in Myeloid Leukemias from Metabolic Stress in the Bone Marrow. Cell Stem Cell.

[113] Kishton R.J., Barnes C.E., Nichols A.G., Cohen S., Gerriets V.A., Siska P.J., Macintyre A.N., Goraksha-Hicks P., de Cubas A.A., Liu T. (2016). AMPK Is Essential to Balance Glycolysis and Mitochondrial Metabolism to Control T-ALL Cell Stress and Survival. Cell Metab..

[114] Lagadinou E.D., Sach A., Callahan K., Rossi R.M., Neering S.J., Minhajuddin M., Ashton J.M., Pei S., Grose V., O’Dwyer K.M. (2013). BCL-2 Inhibition Targets Oxidative Phosphorylation and Selectively Eradicates Quiescent Human Leukemia Stem Cells. Cell Stem Cell.

[115] Tohda S. (2014). NOTCH Signaling Roles in Acute Myeloid Leukemia Cell Growth and Interaction with other Stemness-related Signals. Anticancer Res..

[116] Tavor S., Petit I. (2010). Can inhibition of the SDF-1/CXCR4 axis eradicate acute leukemia?. Semin Cancer Biol..

[117] Morath I., Hartmann T.N., Orian-Rousseau V. (2016). CD44: More than a mere stem cell marker. Int. J. Biochem Cell Biol..

[118] Kremer K.N., Dudakovic A., McGee-Lawrence M.E., Philips R.L., Hess A.D., Smith B.D., van Wijnen A.J., Karp J.E., Kaufmann S.H., Westendorf J.J. (2014). Osteoblasts protect AML cells from SDF-1-induced apoptosis. J. Cell. Biochem..

[119] Sterner R.M., Kremer K.N., Dudakovic A., Westendorf J.J., van Wijnen A.J., Hedin K.E. (1950). Tissue-Nonspecific Alkaline Phosphatase Is Required for MC3T3 Osteoblast-Mediated Protection of Acute Myeloid Leukemia Cells from Apoptosis. J. Immunol..

[120] Kremer K.N., Dudakovic A., Hess A.D., Smith B.D., Karp J.E., Kaufmann S.H., Westendorf J.J., van Wijnen A.J., Hedin K.E. (2015). Histone Deacetylase Inhibitors Target the Leukemic Microenvironment by Enhancing a Nherf1-Protein Phosphatase 1α-TAZ Signaling Pathway in Osteoblasts. J. Biol. Chem..

[121] Shen Z.H., Zeng D.F., Kong P.Y., Ma Y.Y., Zhang X. (2016). AMD3100 and G-CSF disrupt the cross-talk between leukemia cells and the endosteal niche and enhance their sensitivity to chemotherapeutic drugs in biomimetic polystyrene scaffolds. Blood Cells Mol. Dis..

[122] Dong-Feng Z., Ting L., Yong Z., Cheng C., Xi Z., Pei-Yan K. (2014). The TPO/c-MPL pathway in the bone marrow may protect leukemia cells from chemotherapy in AML Patients. Pathol. Oncol. Res..

[123] Kumar B., Garcia M., Weng L., Jung X., Murakami J.L., Hu X., McDonald T., Lin A., Kumar A.R., DiGiusto D.L. (2018). Acute myeloid leukemia transforms the bone marrow niche into a leukemia-permissive microenvironment through exosome secretion. Leukemia.

[124] Krevvata M., Silva B.C., Manavalan J.S., Galan-Diez M., Kode A., Matthews B.G., Park D., Zhang C.A., Galili N., Nickolas T.L. (2014). Inhibition of leukemia cell engraftment and disease progression in mice by osteoblasts. Blood.

[125] Frisch B.J., Ashton J.M., Xing L., Becker M.W., Jordan C.T., Calvi L.M. (2012). Functional inhibition of osteoblastic cells in an in vivo mouse model of myeloid leukemia. Blood.

[126] Tomasoni C., Arsuffi C., Donsante S., Corsi A., Riminucci M., Biondi A., Pievani A., Serafini M. (2023). AML alters bone marrow stromal cell osteogenic commitment via Notch signaling. Front Immunol..

[127] Vallet S., Pozzi S., Patel K., Vaghela N., Fulciniti M.T., Veiby P., Hideshima T., Santo L., Cirstea D., Scadden D.T. (2011). A novel role for CCL3 (MIP-1α) in myeloma-induced bone disease via osteocalcin downregulation and inhibition of osteoblast function. Leukemia.

[128] Galán-Díez M., Borot F., Ali A.M., Zhao J., Gil-Iturbe E., Shan X., Luo N., Liu Y., Huang X.-P., Bisikirska B. (2022). Subversion of Serotonin Receptor Signaling in Osteoblasts by Kynurenine Drives Acute Myeloid Leukemia. Cancer Discov..

[129] Bowers M., Zhang B., Ho Y., Agarwal P., Chen C.C., Bhatia R. (2015). Osteoblast ablation reduces normal long-term hematopoietic stem cell self-renewal but accelerates leukemia development. Blood.

[130] Boutin L., Arnautou P., Trignol A., Ségot A., Farge T., Desterke C., Soave S., Clay D., Raffoux E., Sarry J.-E. (2020). Mesenchymal stromal cells confer chemoresistance to myeloid leukemia blasts through Side Population functionality and ABC transporter activation. Haematologica.

[131] Takam Kamga P., Dal Collo G., Cassaro A., Bazzoni R., Delfino P., Adamo A., Bonato A., Carbone C., Tanasi I., Bonifacio M. (2020). Small Molecule Inhibitors of Microenvironmental Wnt/β-Catenin Signaling Enhance the Chemosensitivity of Acute Myeloid Leukemia. Cancers.

[132] Carter B.Z., Mak P.Y., Wang X., Tao W., Ruvolo V., Mak D., Mu H., Burks J.K., Andreeff M. (2019). An ARC-Regulated IL1β/Cox-2/PGE2/β-Catenin/ARC Circuit Controls Leukemia-Microenvironment Interactions and Confers Drug Resistance in AML. Cancer Res..

[133] Carter B.Z., Qiu Y.H., Zhang N., Coombes K.R., Mak D.H., Thomas D.A., Ravandi F., Kantarjian H.M., Koller E., Andreeff M. (2011). Expression of ARC (apoptosis repressor with caspase recruitment domain), an antiapoptotic protein, is strongly prognostic in AML. Blood.

[134] You R., Hou D., Wang B., Liu J., Wang X., Xiao Q., Pan Z., Li D., Feng X., Kang L. (2022). Bone marrow microenvironment drives AML cell OXPHOS addiction and AMPK inhibition to resist chemotherapy. J. Leukoc. Biol..

[135] Cho B.S., Kim H.J., Konopleva M. (2017). Targeting the CXCL12/CXCR4 axis in acute myeloid leukemia: From bench to bedside. Korean J. Intern Med..

[136] Sison E.A.R., McIntyre E., Magoon D., Brown P. (2013). Dynamic chemotherapy-induced upregulation of CXCR4 expression: A mechanism of therapeutic resistance in pediatric AML. Mol. Cancer Res..

[137] Fiegl M., Samudio I., Clise-Dwyer K., Burks J.K., Mnjoyan Z., Andreeff M. (2009). CXCR4 expression and biologic activity in acute myeloid leukemia are dependent on oxygen partial pressure. Blood.

[138] Ciciarello M., Corradi G., Forte D., Cavo M., Curti A. (2021). Emerging Bone Marrow Microenvironment-Driven Mechanisms of Drug Resistance in Acute Myeloid Leukemia: Tangle or Chance?. Cancers.

[139] Kim D., Kim J., Yoon J.H., Ghim J., Yea K., Song P., Park S., Lee A., Hong C.-P., Jang M.S. (2014). CXCL12 secreted from adipose tissue recruits macrophages and induces insulin resistance in mice. Diabetologia.

[140] Chen Y., Jacamo R., Konopleva M., Garzon R., Croce C., Andreeff M. (2013). CXCR4 downregulation of let-7a drives chemoresistance in acute myeloid leukemia. J. Clin. Investig..

[141] Karjalainen R., Pemovska T., Popa M., Liu M., Javarappa K.K., Majumder M.M., Yadav B., Tamborero D., Tang J., Bychkov D. (2017). JAK1/2 and BCL2 inhibitors synergize to counteract bone marrow stromal cell-induced protection of AML. Blood.

[142] Rigo A., Gottardi M., Zamò A., Mauri P., Bonifacio M., Krampera M., Damiani E., Pizzolo G., Vinante F. (2010). Macrophages may promote cancer growth via a GM-CSF/HB-EGF paracrine loop that is enhanced by CXCL12. Mol. Cancer.

[143] Sánchez-Martín L., Estecha A., Samaniego R., Sánchez-Ramón S., Vega M.Á., Sánchez-Mateos P. (2011). The chemokine CXCL12 regulates monocyte-macrophage differentiation and RUNX3 expression. Blood.

[144] Babazadeh S., Nassiri S.M., Siavashi V., Sahlabadi M., Hajinasrollah M., Zamani-Ahmadmahmudi M. (2021). Macrophage polarization by MSC-derived CXCL12 determines tumor growth. Cell. Mol. Biol. Lett..

[145] Çelik H., Lindblad K.E., Popescu B., Gui G., Goswami M., Valdez J., DeStefano C., Lai C., Thompson J., Ghannam J.Y. (2020). Highly multiplexed proteomic assessment of human bone marrow in acute myeloid leukemia. Blood Adv..

[146] Keech T., McGirr C., Winkler I.G., Levesque J.P. (2017). Macrophage Involvement in the Response of Acute Myeloid Leukaemia to Chemotherapy. Blood.

[147] Miari K.E., Guzman M.L., Wheadon H., Williams M.T.S. (2021). Macrophages in Acute Myeloid Leukaemia: Significant Players in Therapy Resistance and Patient Outcomes. Front. Cell Dev. Biol..

[148] Vitale I., Manic G., Coussens L.M., Kroemer G., Galluzzi L. (2019). Macrophages and Metabolism in the Tumor Microenvironment. Cell Metab..

[149] Xiao L., Wang Q., Peng H. (2023). Tumor-associated macrophages: New insights on their metabolic regulation and their influence in cancer immunotherapy. Front. Immunol..

[150] Spertini C., Bénéchet A.P., Birch F., Bellotti A., Román-Trufero M., Arber C., Auner H.W., Mitchell R.A., Spertini O., Smirnova T. (2024). Macrophage migration inhibitory factor blockade reprograms macrophages and disrupts prosurvival signaling in acute myeloid leukemia. Cell Death Discov..

[151] Casanova-Acebes M., Pitaval C., Weiss L.A., Nombela-Arrieta C., Chèvre R., A-González N., Kunisaki Y., Zhang D., van Rooijen N., Silberstein L.E. (2013). Rhythmic modulation of the hematopoietic niche through neutrophil clearance. Cell.

[152] Perzolli A., Koedijk J.B., Zwaan C.M., Heidenreich O. (2024). Targeting the innate immune system in pediatric and adult AML. Leukemia.

[153] Mesbahi Y., Trahair T.N., Lock R.B., Connerty P. (2022). Exploring the Metabolic Landscape of AML: From Haematopoietic Stem Cells to Myeloblasts and Leukaemic Stem Cells. Front. Oncol..

[154] Bolandi S.M., Pakjoo M., Beigi P., Kiani M., Allahgholipour A., Goudarzi N., Khorashad J.S., Eiring A.M. (2021). A Role for the Bone Marrow Microenvironment in Drug Resistance of Acute Myeloid Leukemia. Cells.

[155] Stevens B.M., Jones C.L., Pollyea D.A., Culp-Hill R., D’Alessandro A., Winters A., Krug A., Abbott D., Goosman M., Pei S. (2020). Fatty acid metabolism underlies venetoclax resistance in acute myeloid leukemia stem cells. Nat. Cancer.

[156] Carracedo A., Cantley L.C., Pandolfi P.P. (2013). Cancer metabolism: Fatty acid oxidation in the limelight. Nat. Rev. Cancer.

[157] Ye H., Adane B., Khan N., Sullivan T., Minhajuddin M., Gasparetto M., Stevens B., Pei S., Balys M., Ashton J.M. (2016). Leukemic Stem Cells Evade Chemotherapy by Metabolic Adaptation to an Adipose Tissue Niche. Cell Stem Cell.

[158] Itoh T., Fairall L., Amin K., Inaba Y., Szanto A., Balint B.L., Nagy L., Yamamoto K., Schwabe J.W.R. (2008). Structural basis for the activation of PPARγ by oxidized fatty acids. Nat. Struct. Mol. Biol..

[159] Tabe Y., Yamamoto S., Saitoh K., Sekihara K., Monma N., Ikeo K., Mogushi K., Shikami M., Ruvolo V., Ishizawa J. (2017). Bone Marrow Adipocytes Facilitate Fatty Acid Oxidation Activating AMPK and a Transcriptional Network Supporting Survival of Acute Monocytic Leukemia Cells. Cancer Res..

[160] Shafat M.S., Oellerich T., Mohr S., Robinson S.D., Edwards D.R., Marlein C.R., Piddock R.E., Fenech M., Zaitseva L., Abdul-Aziz A. (2017). Leukemic blasts program bone marrow adipocytes to generate a protumoral microenvironment. Blood.

[161] Jaswal J.S., Keung W., Wang W., Ussher J.R., Lopaschuk G.D. (2011). Targeting fatty acid and carbohydrate oxidation—A novel therapeutic intervention in the ischemic and failing heart. Biochim. Biophys. Acta BBA Mol. Cell Res..

[162] Tabe Y., Konopleva M. (2023). Resistance to energy metabolism—Targeted therapy of AML cells residual in the bone marrow microenvironment. Cancer Drug Resist..

[163] Tabe Y., Saitoh K., Yang H., Sekihara K., Yamatani K., Ruvolo V., Taka H., Kaga N., Kikkawa M., Arai H. (2018). Inhibition of FAO in AML co-cultured with BM adipocytes: Mechanisms of survival and chemosensitization to cytarabine. Sci. Rep..

[164] Moschoi R., Imbert V., Nebout M., Chiche J., Mary D., Prebet T., Saland E., Castellano R., Pouyet L., Collette Y. (2016). Protective mitochondrial transfer from bone marrow stromal cells to acute myeloid leukemic cells during chemotherapy. Blood.

[165] Boyiadzis M., Whiteside T.L. (2018). Exosomes in acute myeloid leukemia inhibit hematopoiesis. Curr. Opin. Hematol..

[166] Hornick N.I., Doron B., Abdelhamed S., Huan J., Harrington C.A., Shen R., Cambronne X.A., Chakkaramakkil Verghese S., Kurre P. (2016). AML suppresses hematopoiesis by releasing exosomes that contain microRNAs targeting c-MYB. Sci. Signal.

[167] Chen T., Zhang G., Kong L., Xu S., Wang Y., Dong M. (2019). Leukemia-derived exosomes induced IL-8 production in bone marrow stromal cells to protect the leukemia cells against chemotherapy. Life Sci..

[168] Michelis F.V., Hedley D.W., Malhotra S., Chow S., Loach D., Gupta V., Kim D.D., Kuruvilla J., Lipton J.H., Viswabandya A. (2019). Mobilization of Leukemic Cells Using Plerixafor as Part of a Myeloablative Preparative Regimen for Patients with Acute Myelogenous Leukemia Undergoing Allografting: Assessment of Safety and Tolerability. Biol. Blood Marrow Transpl..

[169] Martínez-Cuadrón D., Boluda B., Martínez P., Bergua J., Rodríguez-Veiga R., Esteve J., Vives S., Serrano J., Vidriales B., Salamero O. (2018). A phase I-II study of plerixafor in combination with fludarabine, idarubicin, cytarabine, and G-CSF (PLERIFLAG regimen) for the treatment of patients with the first early-relapsed or refractory acute myeloid leukemia. Ann. Hematol..

[170] Roboz G.J., Ritchie E.K., Dault Y., Lam L., Marshall D.C., Cruz N.M., Hsu H.-T.C., Hassane D.C., Christos P.J., Ippoliti C. (2018). Phase I trial of plerixafor combined with decitabine in newly diagnosed older patients with acute myeloid leukemia. Haematologica.

[171] Konopleva M., Benton C.B., Thall P.F., Zeng Z., Shpall E., Ciurea S., Kebriaei P., Alousi A., Popat U., Anderlini P. (2015). Leukemia cell mobilization with G-CSF plus plerixafor during busulfan-fludarabine conditioning for allogeneic stem cell transplantation. Bone Marrow Transpl..

[172] Heiblig M., Elhamri M., Thomas X., Plesa A., Raffoux E., Hayette S. (2018). A phase 1 study of chemosensitization with plerixafor plus G-CSF in adults with relapsed acute myeloid leukemia. Leuk. Res..

[173] Cooper T.M., Sison E.A.R., Baker S.D., Li L., Ahmed A., Trippett T., Gore L., Macy M.E., Narendran A., August K. (2017). A phase 1 study of the CXCR4 antagonist plerixafor in combination with high-dose cytarabine and etoposide in children with relapsed or refractory acute leukemias or myelodysplastic syndrome: A Pediatric Oncology Experimental Therapeutics Investigators’ Consortium study (POE 10-03). Pediatr. Blood Cancer.

[174] Borthakur G., Zeng Z., Cortes J.E., Chen H.C., Huang X., Konopleva M., Ravandi F., Kadia T., Patel K.P., Daver N. (2020). Phase 1 study of combinatorial sorafenib, G-CSF, and plerixafor treatment in relapsed/refractory, FLT3-ITD-mutated acute myelogenous leukemia patients. Am. J. Hematol..

[175] Uy G.L., Rettig M.P., Stone R.M., Konopleva M.Y., Andreeff M., McFarland K., Shannon W., Fletcher T.R., Reineck T., Eades W. (2017). A phase 1/2 study of chemosensitization with plerixafor plus G-CSF in relapsed or refractory acute myeloid leukemia. Blood Cancer J..

[176] Uy G.L., Avigan D., Cortes J.E., Becker P.S., Chen R.W., Liesveld J.L., Hewes B., Johns D., Erba H.P. (2011). Safety and Tolerability of Plerixafor in Combination with Cytarabine and Daunorubicin in Patients with Newly Diagnosed Acute Myeloid Leukemia- Preliminary Results From a Phase I Study. Blood.

[177] Boddu P., Borthakur G., Naqvi K., Wierda W.G., Bose P., Jabbour E., Estrov Z., Burger J.A., Alvarado Y., Deshmukh A. (2018). Initial report of a phase I study of LY2510924 with idarubicin and cytarabine (IA) in relapsed/refractory (R/R) AML. J. Clin. Oncol..

[178] Borthakur G., Ofran Y., Tallman M.S., Foran J., Uy G.L., DiPersio J.F., Showel M.M., Shimoni A., Nagler A., Rowe J.M. (2021). BL-8040 CXCR4 antagonist is safe and demonstrates antileukemic activity in combination with cytarabine for the treatment of relapsed/refractory acute myelogenous leukemia: An open-label safety and efficacy phase 2a study. Cancer.

[179] Zhang Y., Saavedra E., Tang R., Gu Y., Lappin P., Trajkovic D., Liu S.-H., Smeal T., Fantin V., De Botton S. (2017). Targeting primary acute myeloid leukemia with a new CXCR4 antagonist IgG1 antibody (PF-06747143). Sci. Rep..

[180] Jaramillo Segura S., Wass M., Schaffrath J., Rieger K., Nogai A., Haenel M., Herbst R., Stoelzel F., Röllig C., Jost E. (2022). Double-Blind, Placebo Controlled, Randomized, Multicenter, Phase II Study to Assess the Efficacy of the High Affinity CXCR4 Inhibitor BL-8040 As Addition to Consolidation Therapy in AML By the SAL and OSHO Leukemia Study Groups. Blood.

[181] Squibb B.-M. (2021). A Phase 1/2, Open-label Randomized Study of Ulocuplumab (BMS-936564) in Combination with Low Dose Cytarabine in Subjects with Newly Diagnosed Acute Myeloid Leukemia. https://clinicaltrials.gov/study/NCT02305563.

[182] Kirschbaum M.H., Synold T., Stein A.S., Tuscano J., Zain J.M., Popplewell L., Karanes C., O'Donnell M.R., Pulone B., Rincon A. (2011). A phase 1 trial dose-escalation study of tipifarnib on a week-on, week-off schedule in relapsed, refractory or high-risk myeloid leukemia. Leukemia.

[183] Karp J.E., Smith B.D., Gojo I., Lancet J.E., Greer J., Klein M., Morris L., Levis M.J., Gore S.D., Wright J.J. (2008). Phase II Trial of Tipifarnib as Maintenance Therapy in First Complete Remission in Adults with Acute Myelogenous Leukemia and Poor-Risk Features. Clin. Cancer Res..

[184] Muus P., Langemeijer S., van Bijnen S., Blijlevens N., de Witte T. (2021). A phase I clinical trial to study the safety of treatment with tipifarnib combined with bortezomib in patients with advanced stages of myelodysplastic syndrome and oligoblastic acute myeloid leukemia. Leuk. Res..

[185] Luger S.M., Wang V.X., Rowe J.M., Litzow M.R., Paietta E., Ketterling R.P., Lazarus H., Rybka W.B., Craig M.D., Karp J. (2021). Tipifarnib as maintenance therapy did not improve disease-free survival in patients with acute myelogenous leukemia at high risk of relapse: Results of the phase III randomized E2902 trial. Leuk. Res..

[186] Zimmerman T.M., Harlin H., Odenike O.M., Berk S., Sprague E., Karrison T., Stock W., Larson R.A., Ratain M.J., Gajewski T.F. (2004). Dose-Ranging Pharmacodynamic Study of Tipifarnib (R115777) in Patients With Relapsed and Refractory Hematologic Malignancies. J. Clin. Oncol..

[187] Gualberto A., Scholz C., Janes M.R., Kessler L., Raza A. (2017). The CXCL12/CXCR4 Pathway As a Potential Target of Tipifarnib in Acute Myeloid Leukemia and Myelodysplastic Syndromes. Blood.

[188] Gualberto A., Scholz C., Mishra V., Janes M.R., Kessler L., Cutsem E.V., Ho A.L., Witzig T. (2019). Abstract CT191: Mechanism of action of the farnesyltransferase inhibitor, tipifarnib, and its clinical applications. Cancer Res..

[189] Cummins K.D., Frey N.V., Nelson A.M., Schmidt A.H., Luger S.M., Isaacs R., Lacey S.F., Hexner E.O., Melenhorst J.J., June C.H. (2017). Treating Relapsed/Refractory (RR) AML with Biodegradable Anti-CD123 CAR Modified T Cells. Blood.

[190] Malik S., Westcott J.M., Brekken R.A., Burrows F.J. (2022). CXCL12 in Pancreatic Cancer: Its Function and Potential as a Therapeutic Drug Target. Cancers.

[191] Harousseau J.L., Martinelli G., Jedrzejczak W.W., Brandwein J.M., Bordessoule D., Masszi T., Ossenkoppele G.J., Alexeeva J.A., Beutel G., Maertens J. (2009). A randomized phase 3 study of tipifarnib compared with best supportive care, including hydroxyurea, in the treatment of newly diagnosed acute myeloid leukemia in patients 70 years or older. Blood.

[192] Erba H.P., Othus M., Walter R.B., Kirschbaum M.H., Tallman M.S., Larson R.A., Slovak M.L., Kopecky K.J., Gundacker H.M., Appelbaum F.R. (2014). Four different regimens of farnesyltransferase inhibitor tipifarnib in older, untreated acute myeloid leukemia patients: North American Intergroup Phase II study SWOG S0432. Leuk. Res..

[193] Lancet J.E., Gojo I., Gotlib J., Feldman E.J., Greer J., Liesveld J.L., Bruzek L.M., Morris L., Park Y., Adjei A.A. (2006). A phase 2 study of the farnesyltransferase inhibitor tipifarnib in poor-risk and elderly patients with previously untreated acute myelogenous leukemia. Blood.

[194] Jabbour E., Kantarjian H., Ravandi F., Garcia-Manero G., Estrov Z., Verstovsek S., O'Brien S., Faderl S., Thomas D.A., Wright J.J. (2011). A phase 1-2 study of a farnesyltransferase inhibitor, tipifarnib, combined with idarubicin and cytarabine for patients with newly diagnosed acute myeloid leukemia and high-risk myelodysplastic syndrome. Cancer.

[195] Karp J.E., Flatten K., Feldman E.J., Greer J.M., Loegering D.A., Ricklis R.M., Morris L.E., Ritchie E., Smith B.D., Ironside V. (2009). Active oral regimen for elderly adults with newly diagnosed acute myelogenous leukemia: A preclinical and phase 1 trial of the farnesyltransferase inhibitor tipifarnib (R115777, Zarnestra) combined with etoposide. Blood.

[196] Karp J.E., Vener T.I., Raponi M., Ritchie E.K., Smith B.D., Gore S.D., Morris L.E., Feldman E.J., Greer J.M., Malek S. (2012). Multi-institutional phase 2 clinical and pharmacogenomic trial of tipifarnib plus etoposide for elderly adults with newly diagnosed acute myelogenous leukemia. Blood.

[197] Brandwein J.M., Leber B.F., Howson-Jan K., Schimmer A.D., Schuh A.C., Gupta V., Yee K.W.L., Wright J., Moore M., MacAlpine K. (2009). A phase I study of tipifarnib combined with conventional induction and consolidation therapy for previously untreated patients with acute myeloid leukemia aged 60 years and over. Leukemia.

[198] Kovacsovics T.J., Mims A., Salama M.E., Pantin J., Rao N., Kosak K.M., Ahorukomeye P., Glenn M.J., Deininger M.W.N., Boucher K.M. (2018). Combination of the low anticoagulant heparin CX-01 with chemotherapy for the treatment of acute myeloid leukemia. Blood Adv..

[199] Rao N.V., Argyle B., Xu X., Reynolds P.R., Walenga J.M., Prechel M., Prestwich G.D., MacArthur R.B., Walters B.B., Hoidal J.R. (2010). Low anticoagulant heparin targets multiple sites of inflammation, suppresses heparin-induced thrombocytopenia, and inhibits interaction of RAGE with its ligands. Am. J. Physiol.-Cell Physiol..

[200] Huselton E., Rettig M.P., Campbell K., Cashen A.F., DiPersio J.F., Gao F., Jacoby M.A., Pusic I., Romee R., Schroeder M.A. (2021). Combination of dociparstat sodium (DSTAT), a CXCL12/CXCR4 inhibitor, with azacitidine for the treatment of hypomethylating agent refractory AML and MDS. Leuk. Res..

[201] Chimerix (2024). A Randomized, Double-Blind, Placebo-Controlled Study to Evaluate the Efficacy and Safety of Dociparstat Sodium in Combination with Standard Chemotherapy for the Treatment of Newly Diagnosed Acute Myeloid Leukemia. https://clinicaltrials.gov/study/NCT04571645.

[202] Kovacsovics T., Levy M.Y., Cook R.J., Kolitz J.E., Westervelt P., Donnellan W.B., Stuart R.K., Reagan J.L., Tsai M.L., Wieduwilt M.J. (2019). A randomized phase II trial of CX-01 with standard therapy in elderly patients with acute myeloid leukemia (AML). J. Clin. Oncol..

[203] Bruns I., Lucas D., Pinho S., Ahmed J., Lambert M.P., Kunisaki Y., Scheiermann C., Schiff L., Poncz M., Bergman A. (2014). Megakaryocytes regulate hematopoietic stem cell quiescence through CXCL4 secretion. Nat. Med..

[204] Erbani J., Tay J., Barbier V., Levesque J.P., Winkler I.G. (2020). Acute Myeloid Leukemia Chemo-Resistance Is Mediated by E-selectin Receptor CD162 in Bone Marrow Niches. Front. Cell Dev. Biol..

[205] Barbier V., Erbani J., Fiveash C., Davies J.M., Tay J., Tallack M.R., Lowe J., Magnani J.L., Pattabiraman D.R., Perkins A.C. (2020). Endothelial E-selectin inhibition improves acute myeloid leukaemia therapy by disrupting vascular niche-mediated chemoresistance. Nat. Commun..

[206] DeAngelo D.J., Jonas B.A., Liesveld J.L., Bixby D.L., Advani A.S., Marlton P., Magnani J.L., Thackray H.M., Feldman E.J., O’Dwyer M.E. (2022). Phase 1/2 study of uproleselan added to chemotherapy in patients with relapsed or refractory acute myeloid leukemia. Blood.

[207] Almanza Huante E., Bataller A., Hammond D.E., Chien K.S., DiNardo C.D., Short N.J., Maiti A., Montalban-Bravo G., Ravandi F., Garcia-Manero G. (2023). Uproleselan Added to Cladribine Plus Low Dose Cytarabine (LDAC) in Patients with Treated Secondary Acute Myeloid Leukemia (TS-AML). Blood.

[208] DeAngelo D.J., Schuh A.C., Jonas B.A., Becker P.S., Advani A.S., Uy G.L., Krawczyk J., Erba H.P., Mannis G.N., Fong C.Y. (2024). Efficacy and Safety of Uproleselan Combined with Chemotherapy Vs. Chemotherapy Alone in Relapsed/Refractory Acute Myeloid Leukemia: Findings from an International Phase 3 Trial. Blood.

[209] Benoit Y.D., Mitchell R.R., Risueño R.M., Orlando L., Tanasijevic B., Boyd A.L., Aslostovar L., Salci K.R., Shapovalova Z., Russell J. (2017). Sam68 Allows Selective Targeting of Human Cancer Stem Cells. Cell Chem. Biol..

[210] Rajabi H., Ahmad R., Jin C., Kosugi M., Alam M., Joshi M.D., Kufe D. (2012). MUC1-C Oncoprotein Induces TCF7L2 Transcription Factor Activation and Promotes Cyclin D1 Expression in Human Breast Cancer Cells. J. Biol. Chem..

[211] Huang L., Chen D., Liu D., Yin L., Kharbanda S., Kufe D. (2005). MUC1 Oncoprotein Blocks Glycogen Synthase Kinase 3β–Mediated Phosphorylation and Degradation of β-Catenin. Cancer Res..

[212] Kufe D.W. (2013). MUC1-C oncoprotein as a target in breast cancer: Activation of signaling pathways and therapeutic approaches. Oncogene.

[213] Rizzieri D.A., Cooley S., Odenike O., Moonan L., Chow K.H., Jackson K., Wang X., Brail L., Borthakur G. (2016). An open-label phase 2 study of glycogen synthase kinase-3 inhibitor LY2090314 in patients with acute leukemia. Leuk. Lymphoma.

[214] Lee J.H., Faderl S., Pagel J.M., Jung C.W., Yoon S.S., Pardanani A.D., Becker P.S., Lee H., Choi J., Lee K. (2020). Phase 1 study of CWP232291 in patients with relapsed or refractory acute myeloid leukemia and myelodysplastic syndrome. Blood Adv..

[215] Liegel J., Rosenblatt J., Stone R.M., McMasters M., Levine J.D., Nahas M., Joyce R., Jain S., DeAngelo D.J., Garcia J.S. (2017). Phase I/Ib Trial of the MUC1 Inhibitor GO-203-2C Alone and in Combination with Decitabine for Acute Myeloid Leukemia. Blood.

[216] Insel Gruppe A.G., University Hospital Bern (2018). Epacadostat with Cladribine and Cytarabine (ECC) in Relapsed/Refractory AML Patients Fit for Intensive Chemotherapy; a Phase I Study. https://clinicaltrials.gov/study/NCT03491579.

[217] Insel Gruppe A.G., University Hospital Bern (2018). Epacadostat with Idarubicin and Cytarabine (EIC) for First-Line Treatment of AML Patients Fit for Intensive Chemotherapy; a Phase I Study. https://clinicaltrials.gov/study/NCT03444649.

[218] Emadi A., Duong V.H., Pantin J., Imran M., Koka R., Singh Z., Sausville E.A., Law J.Y., Lee S.T., Shi H. (2018). Indoximod Combined with Standard Induction Chemotherapy Is Well Tolerated and Induces a High Rate of Complete Remission with MRD-Negativity in Patients with Newly Diagnosed AML: Results from a Phase 1 Trial. Blood.

[219] Yao Y., Liu Y.-Y., Li J.-F., Chen Y.-S., Shi L., Shen Y., Yang L.-L., Yang Q. (2025). Indoleamine 2,3-dioxygenase 1 alters the proportions of B cell subpopulations in the microenvironment of acute myeloid leukemia. Mol. Biomed..

[220] Reikvam H., Brenner A.K., Hagen K.M., Liseth K., Skrede S., Hatfield K.J., Bruserud Ø. (2015). The cytokine-mediated crosstalk between primary human acute myeloid cells and mesenchymal stem cells alters the local cytokine network and the global gene expression profile of the mesenchymal cells. Stem Cell Res..

[221] Zhang Y., Guo H., Zhang Z., Lu W., Zhu J., Shi J. (2022). IL-6 promotes chemoresistance via upregulating CD36 mediated fatty acids uptake in acute myeloid leukemia. Exp. Cell Res..

[222] Peterlin P., Garnier A., Le Bourgeois A., Guillaume T., Le Bris Y., Theisen O., Béné M.C., Eveillard M., Rimbert M., Jullien M. (2023). Tocilizumab in combination with a standard induction chemotherapy in acute myeloid leukaemia patients (TOCILAM study): A single-centre, single-arm, phase 1 trial. eClinicalMedicine.

[223] Sadarangani A., Pineda G., Lennon K.M., Chun H.J., Shih A., Schairer A.E., Court A.C., Goff D.J., Prashad S.L., Geron I. (2015). GLI2 inhibition abrogates human leukemia stem cell dormancy. J. Transl. Med..

[224] Fukushima N., Minami Y., Kakiuchi S., Kuwatsuka Y., Hayakawa F., Jamieson C., Kiyoi H., Naoe T. (2016). Small-molecule Hedgehog inhibitor attenuates the leukemia-initiation potential of acute myeloid leukemia cells. Cancer Sci..

[225] Trowbridge J.J., Scott M.P., Bhatia M. (2006). Hedgehog modulates cell cycle regulators in stem cells to control hematopoietic regeneration. Proc. Natl. Acad. Sci. USA.

[226] Roche H.-L. (2015). A Phase Ib/Ii Study to Evaluate the Safety and Efficacy of Vismodegib in Relapsed/Refractory Acute Myelogenous Leukemia (Aml) and Relapsed/Refractory High-Risk Myelodysplastic Syndrome (Mds). https://clinicaltrials.gov/study/NCT01880437.

[227] Pharmaceuticals N. (2016). A Phase II Multi-Center, Open Label, Randomized Study to Assess Safety and Efficacy of Two Different Schedules of Oral LDE225 in Adult Patients with Relapsed/Refractory or Untreated Elderly Patients with Acute Leukemia. https://clinicaltrials.gov/study/NCT01826214.

[228] Martinelli G., Oehler V.G., Papayannidis C., Courtney R., Shaik M.N., Zhang X., O’Connell A., McLachlan K.R., Zheng X., Radich J. (2015). Treatment with PF-04449913, an oral smoothened antagonist, in patients with myeloid malignancies: A phase 1 safety and pharmacokinetics study. Lancet Haematol..

[229] Heuser M., Smith B.D., Fiedler W., Sekeres M.A., Montesinos P., Leber B., Merchant A., Papayannidis C., Pérez-Simón J.A., Hoang C.J. (2021). Clinical benefit of glasdegib plus low-dose cytarabine in patients with de novo and secondary acute myeloid leukemia: Long-term analysis of a phase II randomized trial. Ann. Hematol..

[230] Savona M.R., Pollyea D.A., Stock W., Oehler V.G., Schroeder M.A., Lancet J., McCloskey J., Kantarjian H.M., Ma W.W., Shaik M.N. (2018). Phase Ib Study of Glasdegib, a Hedgehog Pathway Inhibitor, in Combination with Standard Chemotherapy in Patients with AML or High-Risk MDS. Clin. Cancer Res. Off. J. Am. Assoc. Cancer Res..

[231] Sekeres M.A., Montesinos P., Novak J., Wang J., Jeyakumar D., Tomlinson B., Mayer J., Jou E., Robak T., Taussig D.C. (2023). Glasdegib plus intensive or non-intensive chemotherapy for untreated acute myeloid leukemia: Results from the randomized, phase 3 BRIGHT AML 1019 trial. Leukemia.

[232] Sekeres M.A., Schuster M., Joris M., Krauter J., Maertens J., Breems D., Gyan E., Kovacsovics T., Verma A., Vyas P. (2022). A phase 1b study of glasdegib + azacitidine in patients with untreated acute myeloid leukemia and higher-risk myelodysplastic syndromes. Ann. Hematol..

[233] Tibes R., Kosiorek H.E., Dueck A.C., Palmer J., Sproat L., Bogenberger J., Hashmi S., Mesa R., Hogan W., Litzow M.R. (2023). Phase 1/1b study of azacitidine and hedgehog pathway inhibitor sonidegib in patients with myeloid neoplasms. Cancer.

[234] Assouline S., Gasiorek J., Bergeron J., Lambert C., Culjkovic-Kraljacic B., Cocolakis E., Zakaria C., Szlachtycz D., Yee K., Borden K.L. (2023). Molecular targeting of the UDP-glucuronosyltransferase enzymes in high-eukaryotic translation initiation factor 4E refractory/relapsed acute myeloid leukemia patients: A randomized phase II trial of vismodegib, ribavirin with or without decitabine. Haematologica.

[235] Cortes J.E., Douglas Smith B., Wang E.S., Merchant A., Oehler V.G., Arellano M., DeAngelo D.J., Pollyea D.A., Sekeres M.A., Robak T. (2018). Glasdegib in combination with cytarabine and daunorubicin in patients with AML or high-risk MDS: Phase 2 study results. Am. J. Hematol..

[236] Ladikou E.E., Sharp K., Simoes F.A., Jones J.R., Burley T., Stott L., Vareli A., Kennedy E., Vause S., Chevassut T. (2025). A Novel In Vitro Model of the Bone Marrow Microenvironment in Acute Myeloid Leukemia Identifies CD44 and Focal Adhesion Kinase as Therapeutic Targets to Reverse Cell Adhesion-Mediated Drug Resistance. Cancers.

[237] Wang X., Mak P.Y., Mu H., Tao W., Rao A., Visweswaran R., Ruvolo V., Pachter J.A., Weaver D.T., Andreeff M. (2020). Combinatorial Inhibition of Focal Adhesion Kinase and BCL-2 Enhances Antileukemia Activity of Venetoclax in Acute Myeloid Leukemia. Mol. Cancer Ther..

[238] Yu Z., Liang Z., Yao X., Ji Y., Yang D., Zhai Y. (2024). APG-2449, a Novel Focal Adhesion Kinase (FAK) Inhibitor, Exhibits Antileukemic Activity and Enhances Lisaftoclax (APG-2575)-Induced Apoptosis in Acute Myeloid Leukemia (AML). Blood.

[239] Fu D., Jiang H., Long A., Harris E., Guo H., Capitano M.L., Wrangle J., Faust J.R., Gopalakrishnapillai A., Pasupuleti S.K. (2025). Dual targeting of tumoral cells and immune microenvironment by blocking the IL-33/IL1RL1 pathway. Nat. Commun..

